# A computational study of astrocytic glutamate influence on post-synaptic neuronal excitability

**DOI:** 10.1371/journal.pcbi.1006040

**Published:** 2018-04-16

**Authors:** Bronac Flanagan, Liam McDaid, John Wade, KongFatt Wong-Lin, Jim Harkin

**Affiliations:** Intelligent Systems Research Centre, University of Ulster, Magee Campus, Derry~Londonderry, Northern Ireland, United Kingdom; Rush University Medical Center, UNITED STATES

## Abstract

The ability of astrocytes to rapidly clear synaptic glutamate and purposefully release the excitatory transmitter is critical in the functioning of synapses and neuronal circuits. Dysfunctions of these homeostatic functions have been implicated in the pathology of brain disorders such as mesial temporal lobe epilepsy. However, the reasons for these dysfunctions are not clear from experimental data and computational models have been developed to provide further understanding of the implications of glutamate clearance from the extracellular space, as a result of EAAT2 downregulation: although they only partially account for the glutamate clearance process. In this work, we develop an explicit model of the astrocytic glutamate transporters, providing a more complete description of the glutamate chemical potential across the astrocytic membrane and its contribution to glutamate transporter driving force based on thermodynamic principles and experimental data. Analysis of our model demonstrates that increased astrocytic glutamate content due to glutamine synthetase downregulation also results in increased postsynaptic quantal size due to gliotransmission. Moreover, the proposed model demonstrates that increased astrocytic glutamate could prolong the time course of glutamate in the synaptic cleft and enhances astrocyte-induced slow inward currents, causing a disruption to the clarity of synaptic signalling and the occurrence of intervals of higher frequency postsynaptic firing. Overall, our work distilled the necessity of a low astrocytic glutamate concentration for reliable synaptic transmission of information and the possible implications of enhanced glutamate levels as in epilepsy.

## Author summary

The role of astrocytes in the excitability and hyperexcitability of neurons is a subject which has gained a lot of attention, particularly in the pathology of neurological disorders including epilepsy. Although not completely understood, the control of glutamate homeostasis is believed to play a role in paroxysmal neuronal hyperexcitability known to precede seizure activity. We have developed a computational model which explores two of the astrocytic homeostatic mechanisms, namely glutamate clearance and gliotransmission, and connect them with a common controlling factor, astrocytic cytoplasmic glutamate concentration. In our model simulations we demonstrate both a slower clearance rate of synaptic glutamate and enhanced astrocytic glutamate release where cytoplasmic glutamate is elevated, both of which contribute to high frequency neuronal firing and conditions for seizure generation. We also describe a viable role for astrocytes as a “high pass” filter, where astrocytic activation in the form of intracellular calcium oscillations is possible for only a certain range of presynaptic neuronal firing rates, the lower bound of the range being reduced where astrocytic glutamate is elevated. In physiological terms this perhaps indicates not only neuronal but also astrocytic glutamate-mediated excitation in the neural-astrocytic network.

## Introduction

Glutamate is the most abundant excitatory neurotransmitter in the brain [1] and due to its neurotoxic effects [2] glutamate homeostasis must be tightly regulated. This requires that glutamate must, for the most part, be contained intracellularly and that the release of glutamate from both neuronal and non-neuronal sources [3–8] is highly controlled and rapidly removed from extracellular regions, a task which is predominantly carried out by astrocytes [9]. It is considered likely that failure to control glutamate homeostasis is involved in a number of pathologies [10] including mesial temporal lobe epilepsy (MTLE) in which there is a significantly high extracellular glutamate concentration in both the inter-ictal and ictal periods [11].

Astrocytes perform the role of glutamate homeostatic maintenance through a combination of glutamate clearance by excitatory amino-acid transporter EAAT2 (GLT-1) [9] and rapid degradation within the astrocyte largely through the action of glutamine synthetase (GS) [12]. Experimental data suggests that extracellular glutamate increases to a higher concentration and is cleared more slowly in the epileptic than the non-epileptic brain [11], which is at odds with the experimental observation that the EAATs are never overwhelmed [13–14]. High extracellular glutamate levels could lead to hyperexcitability of neurons through over-activation of N-methyl-D-aspartate receptor (NMDA)-mediated receptors [15]. The reasons for the failure of the astrocyte in adequately removing extracellular glutamate are unclear; some studies implicate the reduced expression of EAATs in the epileptic foci [16]. However, other reports suggest no reduction in EAAT expression [17] but instead a marked deficiency in astrocytic enzyme, GS [18–20] in the chronic phase of the syndrome. The latter findings have led to the GS hypothesis of epilepsy [21] in which the loss of this particular enzyme results in increased astrocytic intracellular glutamate [22] affecting the ability of EAATs to clear extracellular glutamate [23] and potentially increasing the effects of gliotransmission [24]. This effect has also been found in neurons [25].

Neuron-astrocyte communication dynamics are described using the concept of the tripartite synapse [3]. The tripartite (glutamatergic) synapse model describes an astrocytic Ca^2+^ elevation in response to presynaptic neuronal firing; the astrocytic Ca^2+^ elevation stimulating the release of astrocytic glutamate (as a gliotransmitter [5,7–8]); the astrocytic-released glutamate activating postsynaptic neuronal N-methyl-D-aspartate-receptors (NMDARs), located extra-synaptically. The activation of extra-synaptic NMDARs induces a slow-inward current (SIC) in the nearby neurons, which has been accredited with the promotion of neuronal synchrony [26] and synaptic plasticity [7,27]. The concept has led to a number of computational models of the tripartite synapse in the functional state [27–31], and applied to describe the dysfunctional, epileptic state [32–36]. These models seek to elucidate the implications of disturbed ionic concentrations, paroxysmal depolarising shift and enhanced synchronisation of neurons believed to underlie epileptogenesis [4,26,37]. In particular, the models of [34–36] describe the influence of astrocytic glutamate homeostatic mechanisms to neuronal behaviour. Each of these models describe a dysfunction in glutamate clearance from the extracellular space as a result of EAAT2 downregulation; described implicitly by an altered decay rate or baseline value [35], an inhibition factor within a Michaelis-Menten equation [34] or altered distance between release and receptor sites [36]. However, they did not explain the impact of increased intracellular glutamate and consequential shift in chemical potential of glutamate across the astrocytic membrane. Hence the description of glutamate clearance has remained incomplete.

In this work, we address this by developing a new explicit model for the EAAT2 transporter which utilises the chemical potential of glutamate and other substrates to describe a driving force for glutamate into the astrocytic compartment, and validate this with experimental data. It is by considering this driving force that we may better understand the limitations and variability of the glutamate clearance process. We describe the clearance of glutamate from the extracellular space (ECS) not only as dependent on extracellular glutamate concentration as in the previous computational models described above, but also more realistically as a function of intracellular astrocytic glutamate and sodium (Na^+^) concentration. We incorporate our EAAT2 model within a modelling framework of gliotransmission described by [27], in which we manipulate the astrocytic glutamate concentration, known to be affected by GS activity [22]. To illustrate the implications of variable glutamate clearance and gliotransmission, we extended a compartmental neuronal model based on the detailed framework of De Pitta and Brunel’s model of gliotransmission [27]. We extended this model by incorporating (1) more explicit glutamate clearance through astrocytic uptake, (2) realistic ionic dynamics across the astrocytic membrane determined by the sodium-potassium ATPase pump (NaK-ATPase) and sodium-calcium exchanger (NCX), (3) the density of glutamate molecules within the astrocytic vesicles as a function of cytoplasmic concentration and (4) a number of neuronal glutamatergic ionotropic receptors and voltage-gated ion channels on the postsynaptic compartment. While we acknowledge that fluctuating extracellular ionic concentrations, in particular elevated potassium (K^+^), would likely affect neuronal excitability, the main objective of this paper is to demonstrate the direct excitability induced by glutamate. In this work, we assume that the extracellular K^+^ concentration was not sufficiently high to affect the reversal potential simplifying the model.

With this more complete model, we illustrate a reliance of rapid glutamate clearance and released gliotransmitter content on the astrocytic glutamate concentration, and relate both of these processes to postsynaptic neuronal activity. Importantly, from our model analysis, we found glutamate astrocytic Ca^2+^ activity more responsive to a wider range of periodic presynaptic neuronal firing when the baseline astrocytic level is high. Therefore, the astrocytic glutamate indirectly acts as a high-pass filter for astrocytic stimulation with variable tolerance.

Overall, we propose a new model of astrocyte-neuron interaction in which astrocytic glutamate concentration is the regulating factor in tripartite synaptic transmission. We find that elevated astrocytic glutamate results in high frequency postsynaptic neuronal firing due to a combination of slower uptake and enhanced Ca^2+^-dependent glutamate release.

## Results

**Fig 1:**
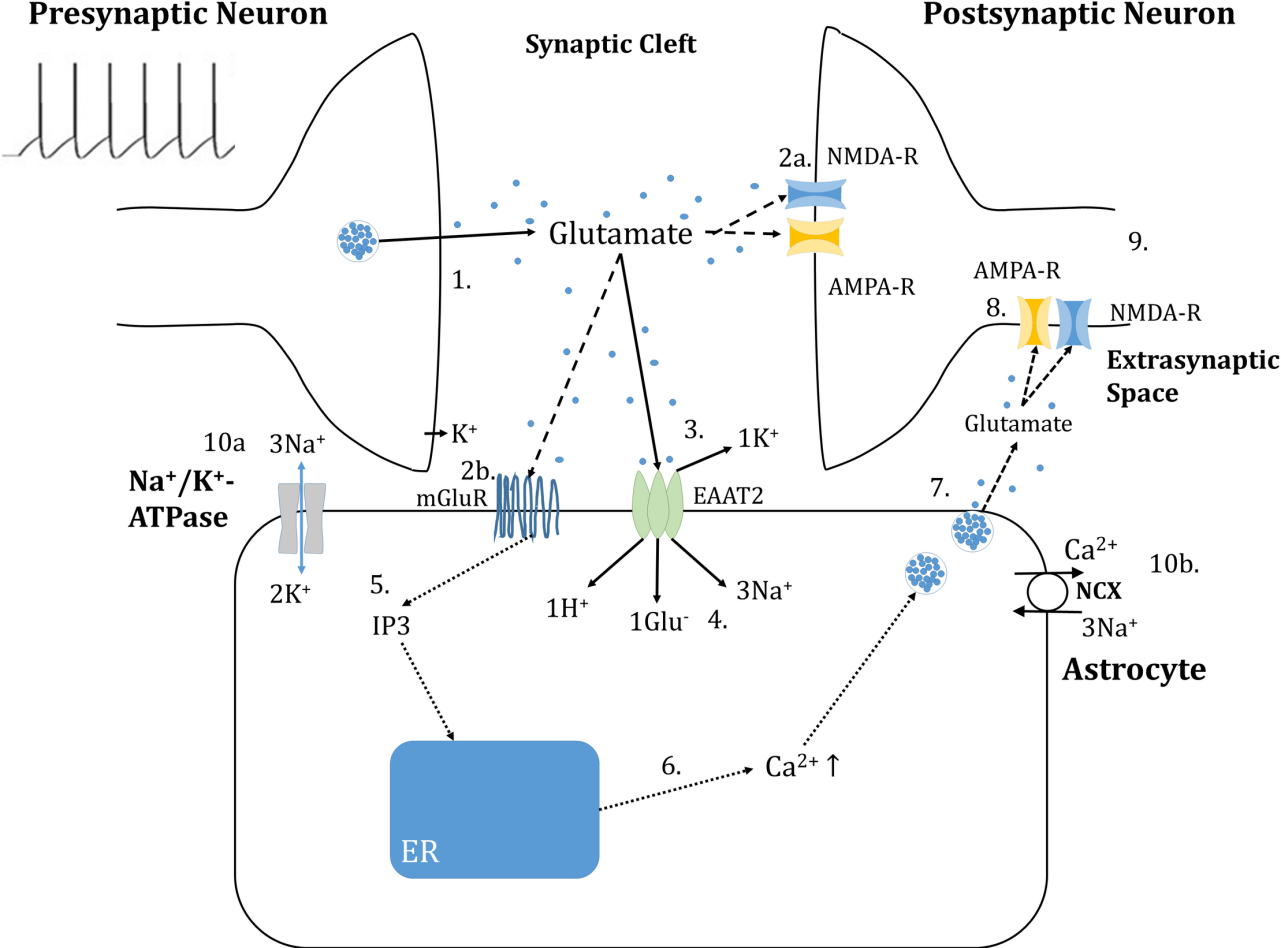
Compartmental model of tripartite glutamatergic synapse. (1) A 10 Hz simulated spike train mimicking *in vivo* spontaneous activity results in a deterministic release of vesicular glutamate and voltage-dependent potassium (K^+^) efflux from the presynaptic neuron into the synaptic cleft: (2a) Glutamate (Glu^-^) activates N-methyl-D-aspartate (NMDA) and α-amino-3-hydroxy-5-methyl-4-isoxazolepropionic acid (AMPA) receptors on the postsynaptic neuron and (2b) Metabotropic glutamate receptors (mGluRs) located on the astrocytic membrane: (3) Glu^-^ is removed from the synaptic cleft compartment by sodium (Na^+^) dependent excitatory amino-acid transporters (EAATs): (4) Glu^-^ and 3Na^+^ enters the astrocytic compartment, the former to be either converted to glutamine or α-ketoglutarate, or packaged into vesicles: (5) Activation of the astrocytic mGluRs results in production of inositol 1, 4, 5-trisphosphate (IP_3_): (6) IP_3_ opens Ca^2+^ channels on the endoplasmic reticulum (ER) allowing an efflux of Ca^2+^ into the cytoplasm in both the soma and perisynaptic process compartments: (7) Ca^2+^ elevation in the process stimulates the release of glutamate vesicles: (8) Astrocytic released glutamate binds to extrasynaptic glutamate receptors: (9) A slow inward current (SIC) is generated in the post-synaptic compartment: Astrocytic homeostatic (10a) Sodium/Potassium pump (NaK-ATPase) removes Na^+^_ast._ and K^+^_syn_ (10b) Sodium-Calcium exchanger (NCX) exchanges 1Ca^2+^ for 3Na^+^ across the membrane.

In order to demonstrate a reliance of glutamate clearance and gliotransmission on astrocytic glutamate, and the impact of both of these processes for postsynaptic activity, a 5-compartmental model was developed within the framework of [27] (Fig 1). Considering the chemical potential of the EAAT2 substrates across the astrocytic membrane and the membrane potential dependent nature of the transporter [38], we developed a model (see supplementary material, S1 Text, for derivation) for the maximal transporter current density (I_EAAT_), in A/m^2^, as

$$I_{EAAT}=-\alpha (e^{-\beta (V_{a}-V_{rev})})$$ (1)

**Fig 2:**
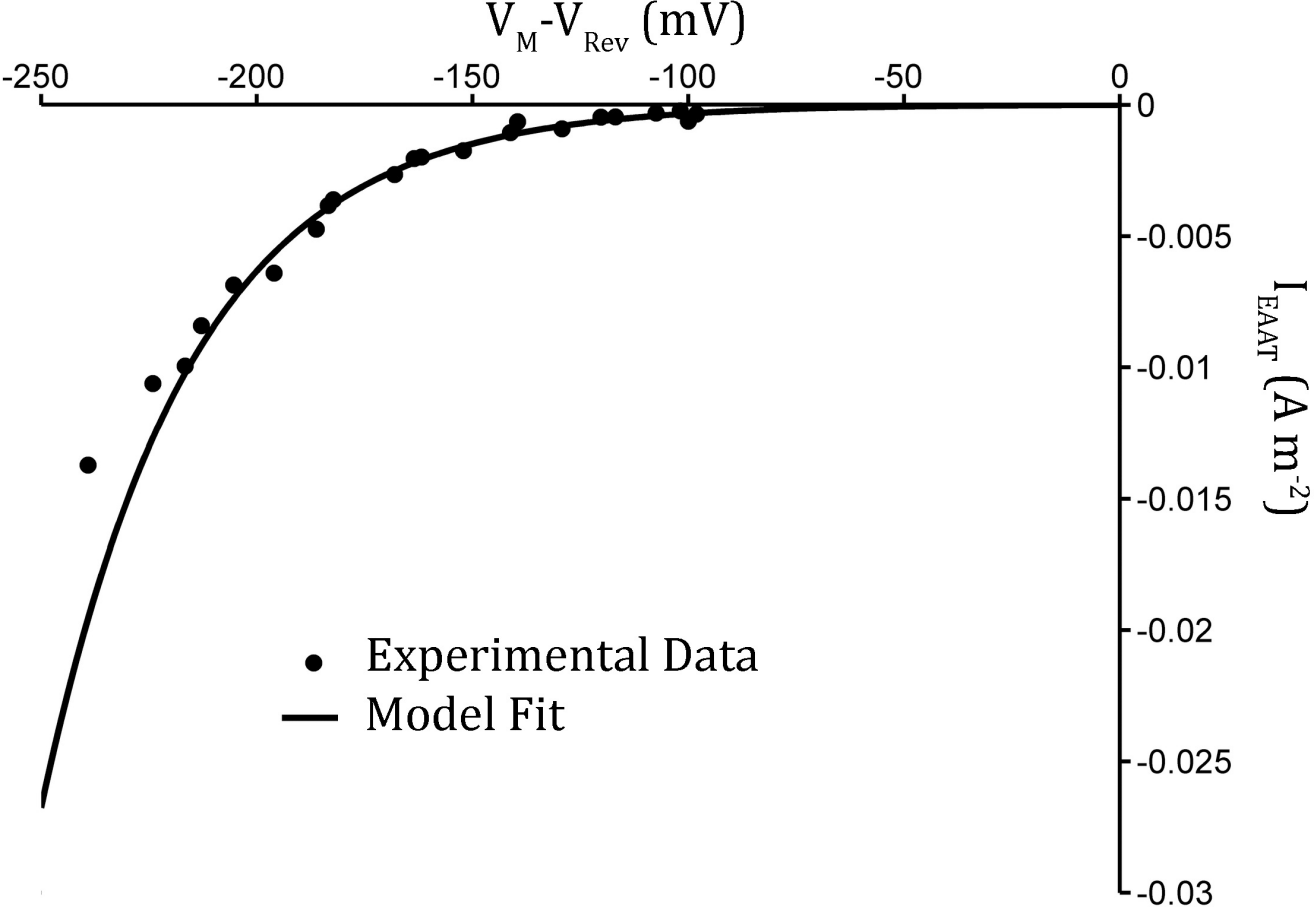
Development of EAAT2 model from experimental data [38]. Computational model for maximal EAAT current density as function of driving force (V-V_rev_) [described in S1 Text]. (Dots: Experimental data [38], line: data fit).

This model was fitted to experimental data [38] to calculate α and β (Fig 2), and utilises the astrocytic membrane potential (V_a_) and transporter reversal potential (V_rev_) described by [38, 39]

$$V_{rev}=\frac{RT}{2F}\ln\left({\left(\frac{{{[Na}^{+}]}_{syn}}{{{[Na}^{+}]}_{ast}}\right)}^{3}\frac{{{[H}^{+}]}_{syn}}{{{[H}^{+}]}_{ast}}\frac{{{[Glu}^{-}]}_{syn}}{{{[Glu}^{-}]}_{ast}}\frac{{{[K}^{+}]}_{ast}}{{{[K}^{+}]}_{syn}}\right)$$ (2)

where [Na^+^]_syn_, [Glu^-^]_syn_, [K^+^]_syn_ and [H^+^]_syn_ are the synaptic (extracellular) concentrations and [Na^+^]_ast_, [Glu^-^]_ast_, [K^+^]_ast_ and [H^+^]_ast_ are astrocytic (intracellular) concentrations of Na^+^, Glu^-^, K^+^, H^+^, respectively, R is the universal gas constant, F is Faraday’s constant and T is the temperature. All parameter values used are given in the Methods section and EAAT model derivation described in supplementary material S1 Text.

We complete the model of the transporter by assuming 1/2 of the current elicited by this transporter is due to glutamate uptake (in one cycle there are net +2 ions transported, including 1 Glu^-^). The current density can be converted to an ionic gradient-supported rate of uptake, V_EAAT,_ with the equation:

$$V_{EAAT}=\frac{1}{2}∙\frac{I_{EAAT}}{F}∙{SA}_{ast}$$ (3)

where SA_ast_ describes the astrocytic membrane surface area at the synapse (see S1 Table).

The results of model simulation are described in two different time-scales: the first is the slower astrocytic response to the presynaptic stimulation and subsequent glutamate release and the second is the much faster post-synaptic neuronal response to astrocytic activation. For each simulation three cases are considered, reflecting three basal astrocytic glutamate concentrations ([Glu^-^]_ast,eq_) of 1.5mM, 5mM and 10mM.

### Neuron-to-astrocyte interaction

**Fig 3:**
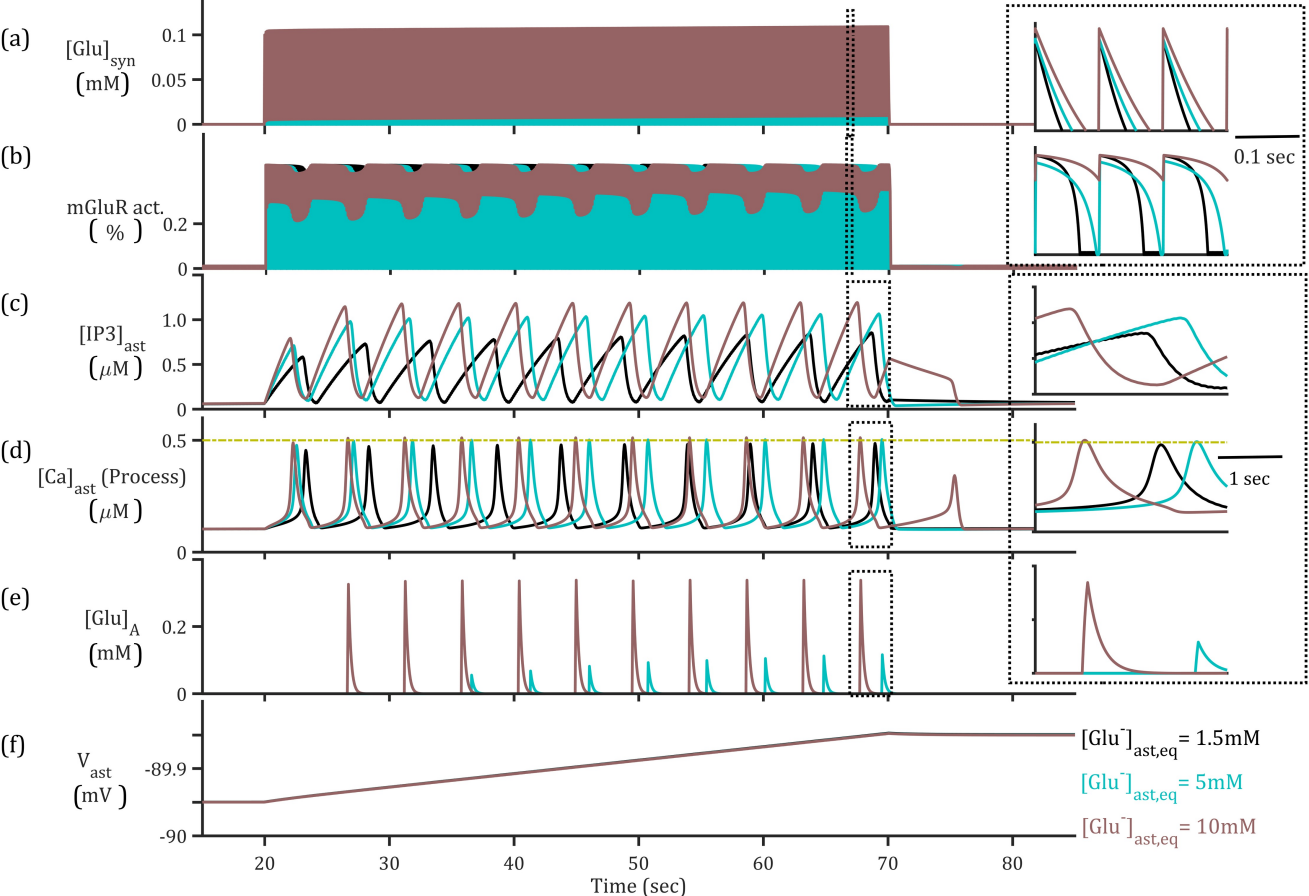
Presynaptic neuron-astrocyte interaction for presynaptic 10 Hz simulation (top) for different basal [Glu]_ast_. (a) The synaptic glutamate concentration resulting from presynaptic release and astrocytic uptake, indicates a longer time course of glutamate in synaptic cleft where [Glu]_ast_ is increased due to slower uptake by EAAT2. Inset: closer view of synaptic glutamate concentration. (b) Higher activation of mGluRs in response to prolonged synaptic glutamate (Inset: closer view of astrocytic mGluRs activation) resulting in (c) perturbation of IP_3_ production and degradation, activating Ca^2+^ ER channels and resulting [Ca^2+^] elevations in the (d) perisynaptic process. (e) The release of glutamate by astrocyte in response to super-threshold Ca^2+^ elevations and enhanced by increased cytosolic [Glu]. (f) Increase in astrocytic membrane potential (V_ast_) as a result of synaptic-driven currents.

**Fig 4:**
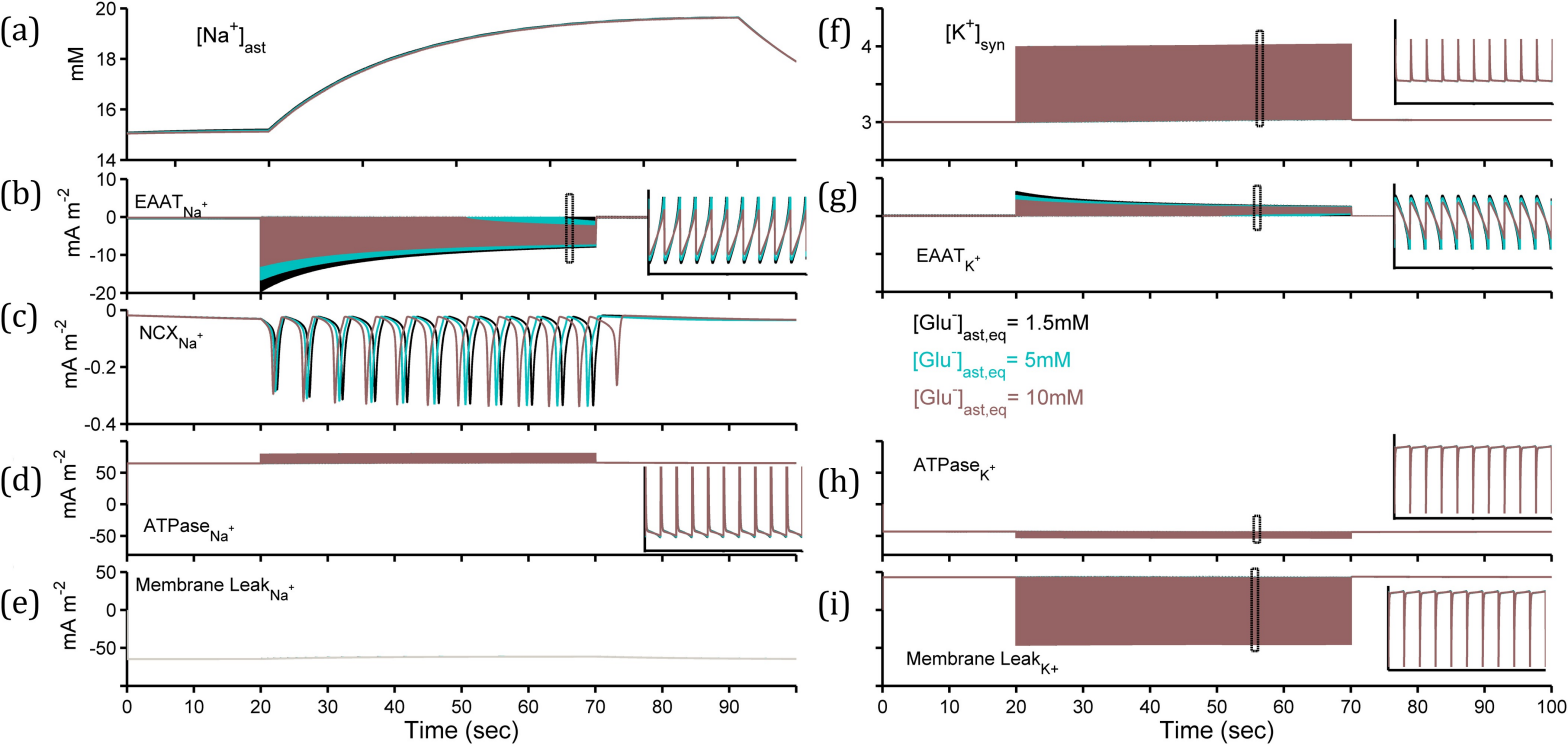
Variable astrocytic sodium ([Na^+^]_ast_) and synaptic potassium ([K^+^]_syn_) concentrations for presynaptic 10Hz simulation. (a) [Na^+^]_ast_ and (b-e) Na^+^ currents generated by EAAT2, NCX, NaK-ATPase & background membrane leak. (f) [K^+^]_syn_ and (g-i) K^+^ currents generated by EAAT2, NaK-ATPase & background membrane leak. (Inset: 1 second closer view).

First we will describe the model’s forward cascading processes from presynaptic to astrocytic activities. The simulated regular spike train of 10 Hz frequency resulted in the deterministic release of Glu^-^ and K^+^ into the synaptic cleft and perturbation of the system. This frequency is chosen to be within the range of *in vivo* cortical neuronal firing behaviour [40]. Synaptic glutamate release activated the astrocytic glutamate transporters, allowing the rapid clearance of glutamate from the synaptic cleft (Fig 3A) and corresponding increase of Na^+^ (Fig 4A). The activity of the glutamate transporters directly affects the concentration of neurotransmitter in the cleft and activation of metabotropic receptors (mGluRs) on the astrocytic membrane (Fig 3B). Activation of astrocytic mGluRs results in the production and ensuing degradation of secondary messenger IP_3_ within the astrocyte (Fig 3C), allowing an efflux of Ca^2+^ from the endoplasmic reticulum (ER) into the astrocytic cytoplasm both in the soma and perisynaptic process (Fig 3D). Elevation of [Ca^2+^] over a threshold value is proposed to be sufficient for glutamatergic gliotransmission (Fig 3E).

### Clearance of glutamate slows as intracellular [Glu]_ast_ is increased

As the presynaptic firing activity is set at 10 Hz for all simulations, any effects observed in the altered uptake of glutamate would be due to the variation of maximal transporter current, mediated in turn by the intracellular (astrocytic) glutamate and Na^+^ concentrations. The 10 Hz presynaptic simulations resulted in similar synaptic maximal glutamate concentrations across the three basal conditions of 1.5mM, 5mM and 10mM (Fig 3A). Upon closer inspection, it reveals a longer time course of glutamate in the synaptic cleft as the basal concentrations of astrocytic glutamate is increased (Fig 3A, inset). The similar maximal values of synaptic glutamate is due to the rapid clearance of glutamate in all three circumstances before the arrival of the next spike. In all cases the glutamate decay rate is within the interval 14–25ms, consistent with experimental observation [41], but with a predicted slower rate where astrocytic glutamate is increased.

In order to ascertain the effects of other EAAT2-substrate ionic dynamics and potential we considered the intracellular Na^+^ and synaptic K^+^ concentrations ([Na^+^]_ast_ and [K^+^]_syn_, respectively) and membrane potential (V_ast_) across the three conditions. The concentrations and V_ast_ were determined not only by EAAT2-mediated currents, but by the ubiquitous sodium-potassium pump (NaK-ATPase), sodium-calcium exchanger (NCX) and concentration-balancing membrane leak currents. In analysing the concentration dynamics (Fig 4) we observed altered individual currents for each ion (Fig 4B–4E & 4G–4I) but ultimately very similar concentrations (Fig 4A & 4B) and V_ast_ (Fig 3F) during the simulation. [Na^+^]_ast_ increased (Fig 4A) due to EAAT2 activation (Fig 4B), and to a lesser degree through NCX (Fig 4C) activation, in keeping with experimental observation [42]. [K^+^]_syn_ was rapidly removed due to a substantial NaK-ATPase current (Fig 4H) for each [Glu^-^]_ast,eq_ considered. Therefore, we reasoned that the delayed removal of synaptic [Glu^-^] must be resulting from a high astrocytic [Glu^-^] level.

Furthermore, we speculate that the occurrence of prolonged glutamate clearance due to presynaptic activity in the healthy brain [41] could compound the problem of glutamate-mediated hyperexcitability and excitotoxicity in the epileptic brain, particularly where glutamate-degrading enzyme is under-expressed in astrocytes [18–20].

### Super-threshold astrocytic Ca^2+^ elevations at the perisynaptic process due to higher basal [Glu]_ast_

**Fig 5:**
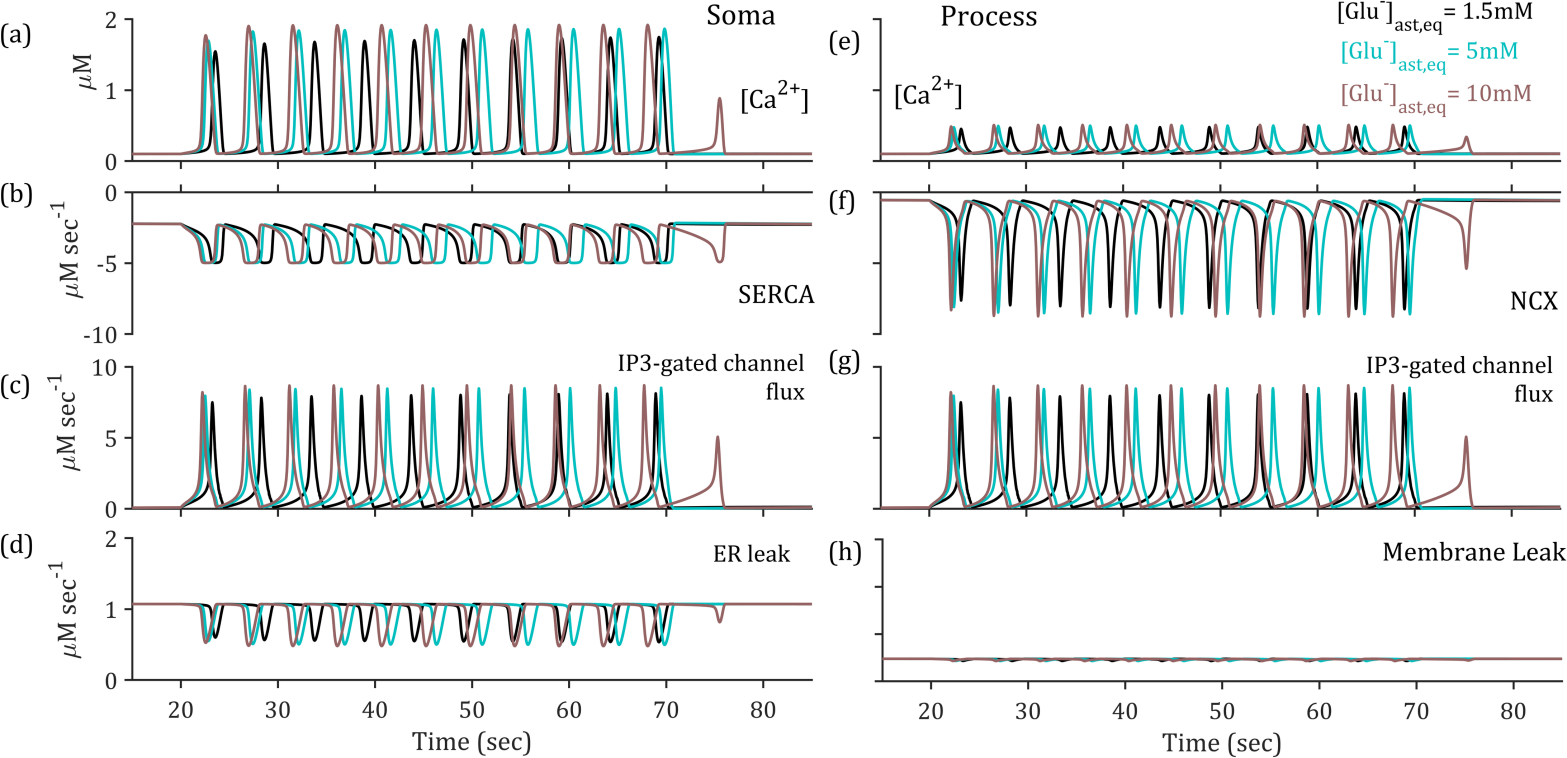
Variable astrocytic calcium ([Ca^2+^]_ast_) concentration in the soma and perisynaptic process for presynaptic 10Hz simultation. (a) [Ca^2+^]_ast,soma_ as determined by (b-d) Sarco/endoplasmic reticulum Ca^2+^-ATPase SERCA pump, IP_3_-gated channels and ER leak fluxes, respectively, and (e) [Ca^2+^]_ast,process_ dynamics as influenced by the (f-h) NCX, synaptic-driven IP_3_ gated channel activation and membrane leak fluxes, respectively.

The time course of synaptic glutamate differs across the three paradigms (Fig 3A inset), highlighted by the altered activation of the mGluRs and the subsequent production of IP_3_ (Fig 3B and 3C). The activation of mGluRs is altered due to prolonged glutamate concentrations in the synaptic compartment (Fig 3B inset), resulting in more oscillatory behaviour due to the interplay of IP_3_ production and degradation triggered by the phospholipase C-β **(**PLC-β) pathway. In turn, the presence of IP_3_ allows a release of Ca^2+^ from ER stores in the astrocytic soma, therefore initiating a [Ca^2+^] elevation. The large flux of [Ca^2+^] from ER stores have been seen to affect concentrations in the far processes of the astrocyte, therefore we model each compartment (soma and process) individually. The model used for IP_3_ and Ca^2+^ dynamics at the soma utilised a proposed mixture of amplitude and frequency modulation (AFM) [43] which meant the Ca^2+^ elevations differed only slightly in terms of amplitude and period (Fig 5A). At the process the [Ca^2+^] is reduced in all cases (Fig 5E) due to efficiency of the NCX (Fig 5F). However, due to the proximity to the proposed threshold for gliotransmission, the increased IP_3_-mediated flux where baseline astrocytic [Glu] is higher (Fig 5G) results in critically increased [Ca^2+^]_process_, thus initiating gliotransmission.

### Stability analysis of astrocytic calcium indicates a high-pass filter effect

The above simulation has so far used a steady 10 Hz presynaptic firing rate which was demonstrated to be sufficient in triggering astrocytic activation in the form of Ca^2+^ oscillations both in the soma and the perisynaptic process (Fig 3D). The Ca^2+^ oscillatory dynamics in this model reflects an interplay between IP_3_-dependent release from the ER and delayed removal of Ca^2+^ at the soma by the SERCA pump and at the process by the NCX. It follows that no change in cytoplasmic [Ca^2+^]_process_ reflects either that there is no IP_3_-dependent activation of Ca^2+^ or a balance between removal and release from the ER. As astrocytic IP_3_-production is prompted by synaptic glutamate and the time course of this glutamate is altered by astrocytic glutamate (Fig 3A), we can observe altered IP_3_ levels and thus astrocytic [Ca^2+^] dynamics (Fig 3C and 3D) as a result of this sustained presence of glutamate in the cleft.

**Fig 6:**
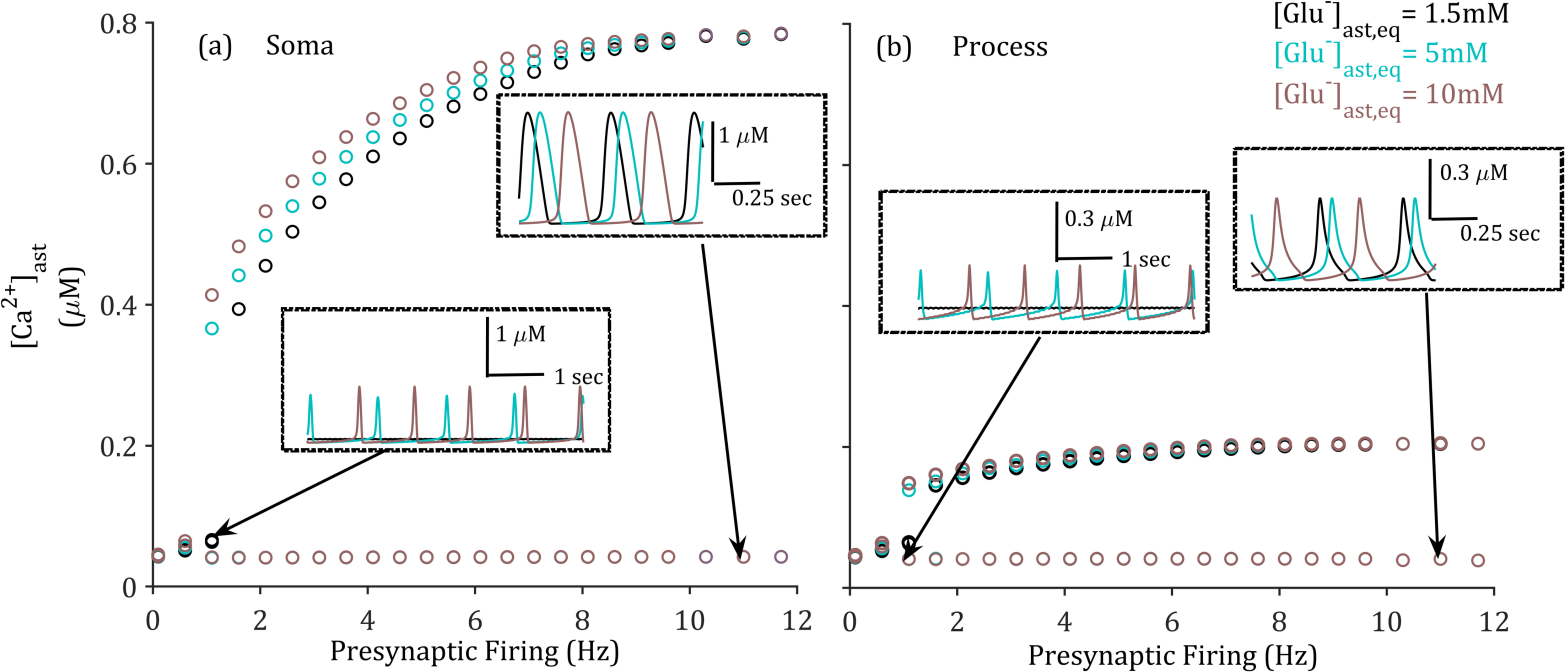
**Stability diagram of astrocytic calcium activity in the (a) soma and (b) perisynaptic process, [Ca**^**2+**^**]**_**ast**_, **on frequency of periodic presynaptic firing activity under different baseline astrocytic level [Glu]**_**ast,eq**_. (o) denotes upper and lower amplitudes of oscillation at steady state. Lower bound of induced oscillatory regime is increased with decreasing [Glu]_ast,eq_. Demonstrates a clear range of input presynaptic firing frequencies which result in Ca^2+^ activation across the three measured [Glu]_ast,eq_. where increasing [Glu]_ast,eq_ correlates with reduction in the lower limit of this range.

Therefore, we now investigate how the system would behave under the gradual increase of presynaptic firing rates, for differing baseline [Glu]_ast_. We assume the presynaptic release of glutamate and K^+^ are deterministic for each simulated spike and all parameters except [Glu]_ast,eq_ are identical. A stability diagram of [Ca^2+^]_ast_ in both the soma and perisynaptic process with respect to periodic presynaptic firing frequency is plotted in Fig 6. The stability diagrams illustrate a lower bound of presynaptic firing frequencies which result in astrocytic Ca^2+^ induced oscillation in both compartments. Note that the frequencies are relatively low, but relevant for typical cortical function [40]. Interestingly, this lower bound of (induced) oscillatory regime can be reduced with higher [Glu]_ast,eq_. In physiological terms this illustrates an enhancement of astrocytic excitability to reduced stimulation with baseline [Glu]_ast,eq_ as a controlling factor, particularly where we also consider the stability of the other ion dynamics for the same protocol (S1 Fig). In other words, the responsiveness of the astrocyte in terms of [Ca^2+^] fluctuations to a certain range of presynaptic firing frequencies behaves similar to a high-pass filter, the limits of which are determined by astrocytic glutamate level.

### The astrocyte to neuron pathway

**Fig 7:**
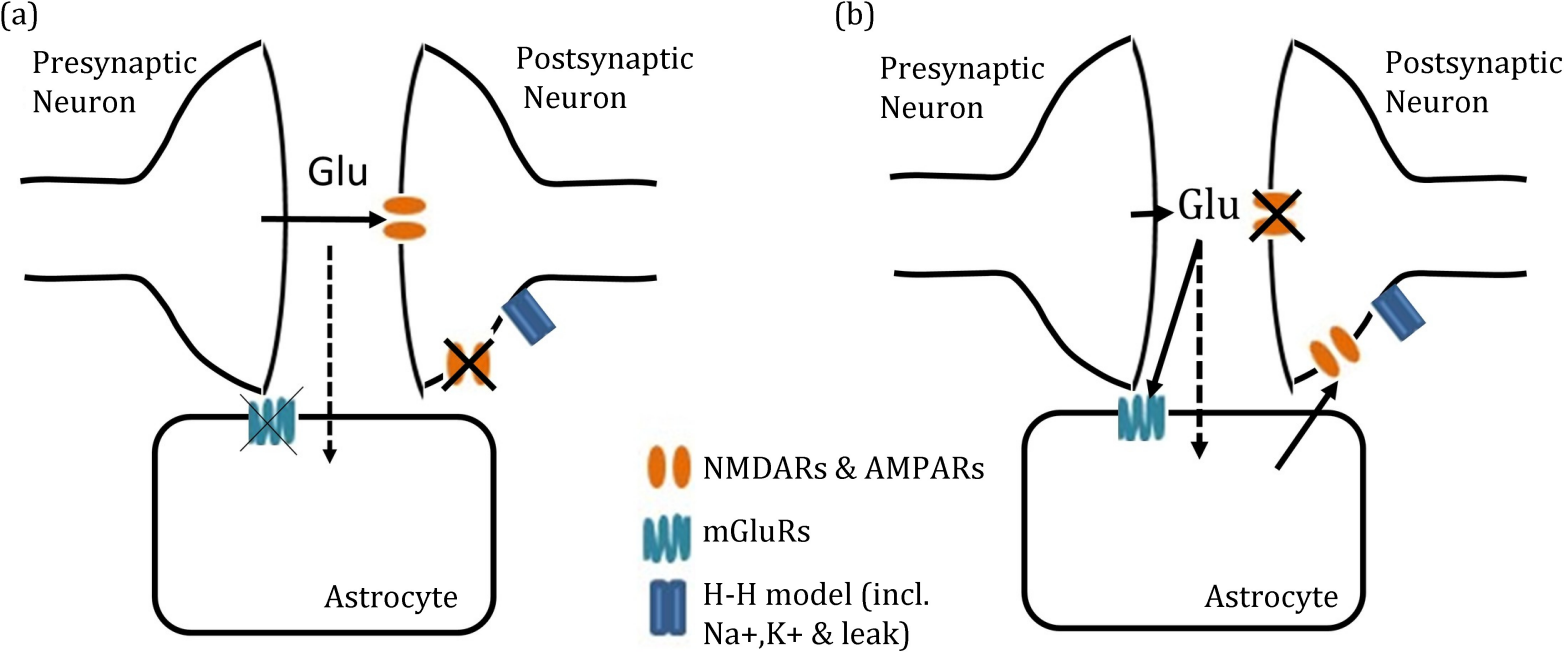
Schematic representations of the two pathways in the model. (a) Direct pre- to post-synaptic neuron transmission only, passive astrocyte responsible for glutamate uptake (dotted line). (b) Indirect pre-to post-synaptic (via astrocyte activation) transmission only.

In our model, the postsynaptic membrane potential is subjected to synaptic currents driven by synaptically-released glutamate, an extra-synaptic SIC driven by astrocyte-released glutamate and intrinsic Na^+^, K^+^ and leak currents. We hypothesised that the slower rate of synaptic glutamate clearance (Fig 3A) combined with enhanced astrocytic-glutamate release (Fig 3G) would lead to high frequency postsynaptic firing provoked by over-activation of synaptic and extra-synaptic ionotropic glutamate AMPA- and NMDA-mediated receptors (AMPARs and NMDARs). In order to consider the impact of both synaptic and extra-synaptic glutamate on the postsynaptic response, we first investigate synaptic currents and SIC independently before considering both simultaneously. These direct and indirect pathways are illustrated in Fig 7.

### Glutamate clearance disruption of synaptic signalling

**Fig 8:**
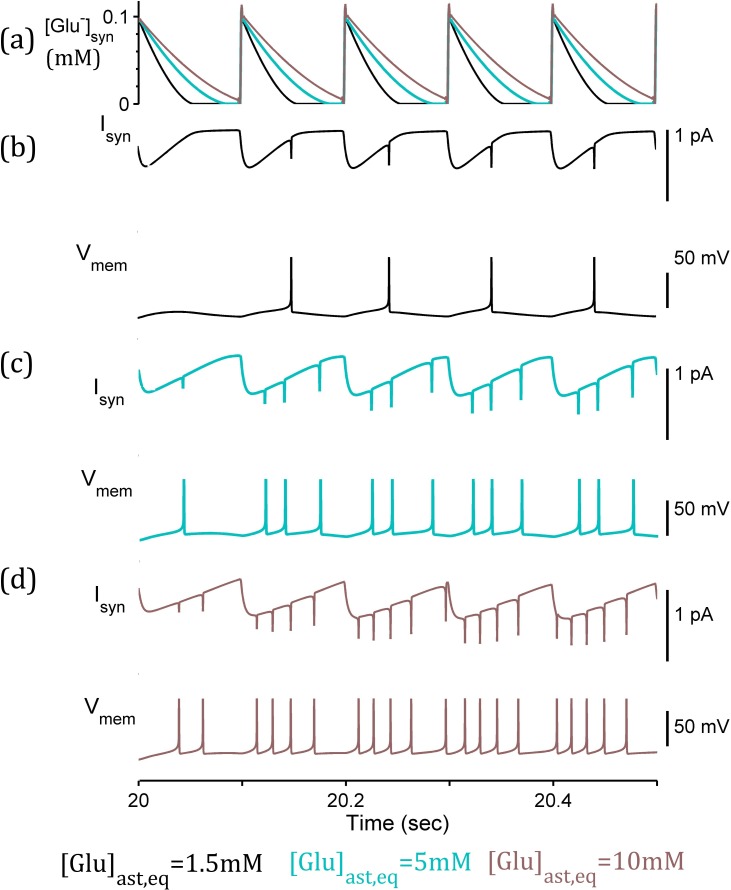
**Postsynaptic activity due to synaptic and intrinsic currents**, triggered by (a) synaptic glutamate [Glu]_syn_ (b-d) simulation with [Glu]_ast,eq_ = 1.5mM, 5mM, and 10mM respectively, synaptic currents (I_syn_) combined AMPA- and NMDA-mediated currents in response to synaptic glutamate, membrane potential (V_m_) of postsynaptic neuron resulting from combination of I_syn_ and voltage-gated currents (Na^+^, K^+^ and leak). Prolonged time course of synaptic glutamate leads to enhanced synaptic currents (I_syn_) and higher frequency postsynaptic firing response (V_m_ depolarisations) as [Glu]_ast,eq_ increases.

We considered the direct neuron-to-neuron signalling through synaptically-released glutamate and subsequent activation of synaptic NMDA and AMPA receptors on the postsynaptic compartment (Fig 7A). As previously demonstrated, the time course of synaptic glutamate is increased as a result of slower uptake when astrocytic [Glu] is increased (Fig 8A). The effects of this alteration are illustrated in Fig 8B–8D, where the [Glu]_syn_-activated NMDA- and AMPA-mediated currents, depolarise the postsynaptic neuron at higher frequencies corresponding to higher [Glu]_ast,eq_.

The progression of increased postsynaptic firing frequencies with increasing astrocytic glutamate concentration suggests an increase in the excitability of synaptic response due to longer time course of glutamate in the synaptic cleft as a result of slower uptake. This in turn leads to prolonged synaptic glutamate concentration, activating a greater fraction of ionotropic receptors and therefore an increased magnitude of synaptic-mediated postsynaptic currents. As the only factor to have been adjusted in this model, we can infer astrocytic glutamate concentration as a controlling factor in the precision of the signal transmission from pre-synaptic to post-synaptic neurons. Hence we would expect downregulated GS to enhance neuronal excitability.

### Post-synaptic neuronal depolarisation due to enhanced gliotransmission

**Fig 9:**
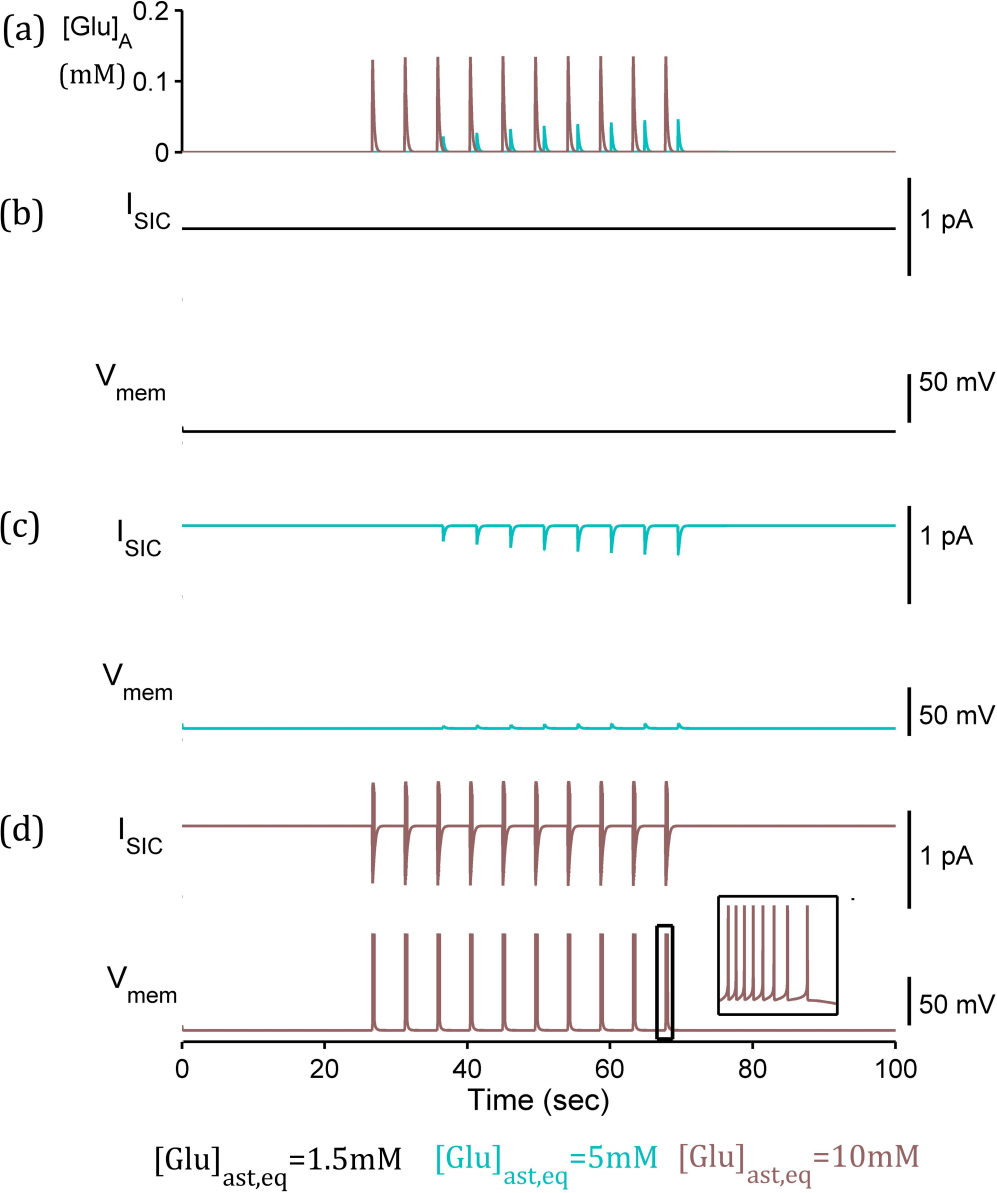
Postsynaptic membrane potential due to SIC and intrinsic currents simulation. (a) extrasynaptic glutamate (G_A_) released by the astrocyte. (b-d) [Glu]_ast,eq_ = 1.5mM, 5mM, and 10mM respectively, (*right* closer view of boxed area for [Glu]_ast,eq_ = 10 mM). SIC (I_sic_) in each case given (*above*) and resulting postsynaptic membrane potential (V_m_) (*below*). Enhanced release of astrocytic glutamate results in stronger and prolonged I_sic_ and subsequent prolonged high-frequency postsynaptic firing (V_m_ depolarisations) due to increasing [Glu]_ast,eq_.

In considering the impact of the SIC, we removed the direct impact of the synaptic glutamate (Fig 7B) resulting in the postsynaptic neuron (Fig 9) displaying intervals of continuous depolarisations (elevated subthreshold membrane potential). This is due to the SIC only where astrocytic, and thus vesicular [Glu], is sufficiently high (Fig 9D). This result promotes the concept of an astrocytic-induced, rather than synaptic-stimulated, postsynaptic firing as demonstrated in [4]. This result is consistent with the experimental results of [44] correlating increased astrocytic glutamate content with increased quantal size of excitatory postsynaptic response.

### Enhanced gliotransmission disrupts synaptic signalling

**Fig 10:**
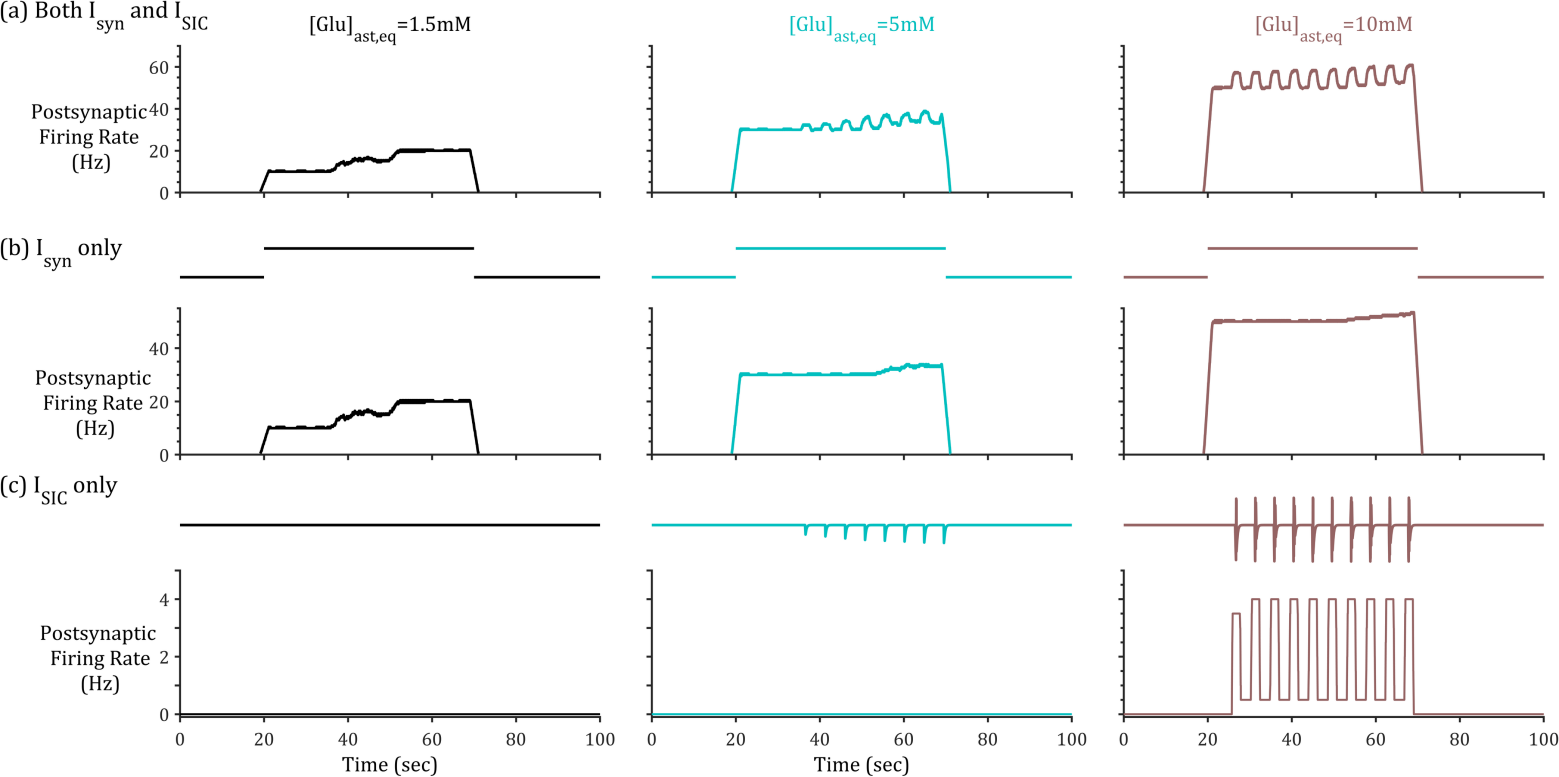
Frequency of postsynaptic firing due to combination of synaptic, extrasynaptic and intrinsic currents. Simulation with [Glu]_ast,eq_ = 1.5mM, 5mM, and 10mM (*left* to *right*). (a) Frequency of postsynaptic firing due to the glutamate release both directly from the presynaptic neuron and from the astrocyte, (b) Frequency of postsynaptic firing due to synaptic glutamate-mediated currents and (c) Frequency of postsynaptic firing due to astrocytic-released-activated currents. Presynaptic activation given as (above b) bar and (above c) SIC. Frequency of postsynaptic firing was calculated using rectangular windowing of length 2 sec, 0.1 overlap. Increasing [Glu]_ast,eq_ results in higher baseline postsynaptic firing to identical presynaptic stimuli determined by longer time course of synaptic glutamate and thus enhanced synaptic-mediated currents. Increasing [Glu]_ast,eq_ also results in longer intervals of high (~60Hz where [Glu]_ast,eq_ = 10mM) frequency, longer lasting postsynaptic depolarisations as a result of enhanced gliotransmission.

Next we analyse the glutamate concentrations in both the synaptic cleft and the astrocyte-released site to determine how the dynamic connections modulate postsynaptic activity. This model used NMDA and AMPA-mediated currents at the synaptic cleft activated by synaptic glutamate (Fig 3A) and the SIC activated by astrocyte-released glutamate (Fig 3E). The model was completed with the same intrinsic currents as above in order to emulate the neuronal response to both synaptic and extra-synaptic activation. The postsynaptic firing activity was analysed using a sliding window to calculate the mean number of spikes over the simulation. The results of this simulation clearly demonstrate both an increased baseline postsynaptic firing response (mediated by synaptic currents (Fig 10B)) from approximately ~10 Hz to ~55 Hz in response to the same uniform presynaptic firing activity where astrocytic glutamate is increased from 1.5mM to 10mM (Fig 10A), and higher frequency intervals due to enhanced gliotransmission (Fig 10C). The maximum postsynaptic firing within these intervals increased from ~20 Hz to ~60 Hz for increased levels of Glu_ast,eq_ from 1.5mM to 10mM.

## Discussion

Excessive concentrations of extracellular glutamate in the region of the epileptic focus [11] appears to implicate astrocyte dysfunction within the pathology of epilepsy. A number of computational models which illustrate both excessive synaptic glutamate and astrocyte-released glutamate have been developed [32–36] to help understand the implications of glutamate excess for neuronal function. This model differs from existing models as it proposes an implicit description of the downregulation of glutamine synthetase in the focal astrocytes [18–20]. We have proposed two probable implications for the downregulation of GS and subsequent increase in astrocytic glutamate concentration [21–22], the disruption to EAAT2 function and thus glutamate clearance from the synaptic cleft, and enhanced gliotransmission as a result of increased glutamate content.

Our model for the maximal current allowed by the EAAT2 arises from principles of thermodynamics and the observation that the transport of Glu^-^ is coupled to the transport of Na^+^, K^+^ and H^+^ which is sufficient to overcome the concentration gradient between extracellular and intracellular glutamate. These observations imply a need for astrocytes to tightly control its intracellular ion concentrations, largely carried out by NaK ATPase pumps [45] and GS [21]. Studies in energetics have proposed that the energetic cost of glutamate uptake is ‘paid’ by metabolism of glutamate through the Krebs cycle [46], however as the production of energy currency adenosine triphosphate (ATP) appears to be activity-driven [47], this alternative pathway would be activated only when the EAAT2s are functioning correctly. Thus as GS is downregulated, astrocytic [Glu^-^] will increase [22] slowing the EAAT2 uptake and thus increasing the time course of synaptic glutamate (Fig 3).

As the model in this paper describes only the transport of ions between the synaptic and astrocytic compartments, the spatial effects of diffusion within compartments have been ignored. In addition, the EAAT2 model developed looks to describe optimal transport rates based on electrochemical gradients, so as a result the binding and unbinding rates of substrates to the EAAT2 protein have not been taken into account. In future work, a more spatially-detailed synaptic model would consider the effects of glutamate-buffering on synaptic concentrations [14,48].

Our model assumes that K^+^ clearance is dominated by NaK-ATPase as this is consistent with *in vivo* experimental data [49], which shows that while inwardly-rectifying K^+^ channel (K_ir_4.1) may play a prominent role for K^+^ uptake at large volume glial processes (e.g. terminal endfeet of retinal Muller cells [50] K^+^ clearance by K_ir_4.1 is much less effective at low volume perisynaptic cradles, which is the present case [45,49,51–52]. Indeed, under physiological conditions the main pathway for K^+^ influx is associated with NaK-ATPase, whereas K_ir_4.1 inward rectifying channels mediate K^+^ efflux which is needed to restore K^+^ gradients in neuronal compartments [51–54]. These observations are consistent with astrocytic K^+^ being re-released via K_ir_4.1 channels at distal synapses after distribution in the astrocytic functional syncytium via gap junctions [18]. Furthermore the sodium-potassium-chloride cotransporter (NKCC) has been ignored in our model as this transporter is widely reported to be only activated at higher K^+^ concentrations (>10mM) [51] which is above the K^+^ concentration simulated in our paper.

EAAT2 function is highly sensitive to fluctuations of intracellular [Na^+^] as is likely in a physiological context [55] and it has been proposed that under pathological conditions where ionic concentrations are disturbed, the glutamate transporters are likely to reverse their direction [56–58]. The transporter model developed in this work allows for disturbance of ionic concentrations up until the point of zero flux, however more experimental data would be required to explain the transport of glutamate in the reversed direction.

It has been demonstrated that neuronal activation by astrocytic-released glutamate was sufficient to cause a paroxysmal depolarising shift similar to those observed in epileptogenesis [4]. Astrocytes are believed to release glutamate through a number of different pathways including Ca^2+^-dependent exocytosis, transporter reversal, the cysteine-glutamate antiporter and a number of volume-controlled channels [58].

Ca^2+^-dependent exocytosis, although widely examined [5–8,24,59] remains a controversial topic [60]. In particular, there is no consensus in *in vivo* studies of the presence of biological components necessary for astrocytic vesicular release, specifically vesicular glutamate transporters (VGLUTs) [59–62]. In the presence of apparent conflicting evidence, Bazargani & Atwell (2016) [63] suggest a highly localised expression of the vesicular protein in astrocytes, supporting the evidence for the heterogeneous nature of astrocytes [59], and this heterogeneity may be the reason for such conflicting views with regards to exocytosis. While the exocytotic nature of astrocytic glutamate release is widely debated, many studies have illustrated astrocytic Ca^2+^-dependent glutamate release [26,64–66] although the mechanism is not fully settled.

This paper considers a Ca^2+^-dependent mechanism as the means of astrocytic glutamate release in our model, whether by exocytosis or otherwise. Astrocytic Ca^2+^-dependent glutamate release has been shown to induce synchronicity of neuronal firing [26] and thus is believed to be a factor in seizure activity [4,44]. In addition, it is plausible that each of the above mechanisms for glutamate release will also contribute to increased glutamate release due to accumulation of glutamate in the cytoplasm, however this would likely increase the background levels of glutamate rather than a transient depolarizing-event such as that demonstrated to be generated by Ca^2+^-dependent glutamate release [5,7,8,24,26,53,58,64–66].

Our model illustrates that an alteration in gliotransmission concentration generates a slow inward current resulting in high frequency activity for a sustained length of time. This concept is also illustrated by [4] in which they were able to reproduce a paroxysmal depolarising shift induced by concentration of astrocytic-released glutamate at the astrocyte-neuron synapse based on effects of spatial phenomena, including diffusion. Our model differs by taking account of the amount of released glutamate as a function of intracellular glutamate concentration. Using this model we are able to simulate both the high frequency activity shown in [4] where astrocytic glutamate is high (~10mM) and much lower frequency activity where astrocytic glutamate is low (~1.5mM).

Our hypothesis for the increased astrocytic vesicular content is based on experimental results which considered neuronal cytoplasmic glutamate concentration and its impact on Ca^2+^-dependent release and quantal size [25]. It has been experimentally demonstrated that astrocytic glutamate release would be affected similarly [44] and that the size of postsynaptic response is heightened as a result of astrocytic cytoplasmic glutamate concentration. This is not necessarily directly due to increased vesicular content, but we propose that vesicular content is moderated by VGLUT protein which perform optimally under acidic conditions; as a result of the accompanying H^+^ influx by EAAT, the intracellular conditions would be likely to become acidic and thus favour glutamate uptake into vesicles [24]. Although we have assumed a linear relationship between astrocytic and vesicular content in this model, it nonetheless illustrates the concept of heightened neuronal response to astrocytic activation.

### Conclusion

In this paper we have proposed a model for glial-neuronal communications which accounts for some of the physiological conditions observed in MTLE: increased extracellular glutamate and non-neuronal provoked intervals of rapid postsynaptic firing. The model presented illustrates both the rate of glutamate uptake from the synaptic cleft following presynaptic release and the concentration of astrocytic-released glutamate by gliotransmission as a function of intracellular astrocytic glutamate concentration. It is likely that this fluctuation of uptake rate occurs in the functional brain as a result of transient ionic perturbations. However, following the downregulation of enzyme activity GS, as in MTLE, there would follow a higher basal concentration of astrocytic glutamate and therefore the EAAT function would be compromised. We have illustrated the effects of the altered synaptic glutamate clearance for both neuronal and astrocytic signalling and report changes to the postsynaptic firing activity as a result. The model also takes into account enhanced gliotransmission for postsynaptic neuronal firing rates and predicts SIC-mediated intervals of higher frequency (up to 65 Hz) firing where the astrocyte-release content was increased. We also reported that lasting synaptic glutamate affects mGluRs-mediated astrocytic [Ca^2+^] activation where the time course of glutamate affects the astrocytic response to lower presynaptic firing stimulation where [Glu]_ast,eq_ is higher. Thus the system behaves as a high pass filter for astrocytic activation, possibly reflecting not only a hyperexcitable neuronal response to prolonged time course of glutamate in the synaptic cleft, but also excessive astrocytic activation, an effect which is far-reaching within the brain by means of the astrocytic network.

## Methods

The model described in this paper consists of two sources of glutamate release: deterministic presynaptic neuron release into a synaptic compartment and Ca^2+^ dependent astrocyte release (gliotransmission) into an extra-synaptic compartment (Fig 1).

### Synaptic glutamate dynamics

We can describe Glu_syn_ as increasing due to presynaptic release (Y_s_) [27] and removed at a rate (V_EAAT_) according to Eq (5). Therefore

$$\frac{d}{dt}{Glu}_{syn}(t)=-\frac{V_{EAAT}(t)}{{Vol}_{syn}}+Y_{s}(t)$$ (4)

Vol_syn_ is the synaptic compartment volume, presynaptic spikes are simulated and at each spike time, (t_k_), glutamate is released from the presynaptic terminal (Y_s_) according to equation

$$Y_{s}(t)=Y_{rel}\delta (t-t_{k})$$ (5)

where Y_rel_ is the concentration of glutamate released (mM) and δ is the Dirac delta function. Glutamate in the synaptic cleft compartment will act as substrate to the EAAT transporter reaction, and thus the EAATs uptake will follow

$$V_{EAAT}(V_{rev}(t),V_{a}(t))=\frac{1}{2F}I_{EAAT}(V_{rev}(t),V_{a}(t))∙{SA}_{ast}$$ (6)

where $I_{EAAT}=-\alpha (e^{-\beta (V_{a}(t)-V_{rev})})$ (Eq 1), α = 1.9767 x 10^−5^ A/m^2^, β = 0.0292 mV^-1^, SA_ast_ is the surface area in question and V_a_ is the astrocytic membrane potential (mV), described below (Eq (13)). The derivation of this equation is detailed in supplementary material S1 Text. The reversal potential, V_rev_, is given by [38–39]

$$V_{rev}(t)=\frac{RT}{2F}\ln\left({\left(\frac{{Na}_{syn}}{{Na}_{ast}(t)}\right)}^{3}(\frac{H_{syn}}{H_{ast}})(\frac{{Glu}_{syn}(t)}{{Glu}_{ast}(t)})(\frac{K_{ast}}{K_{syn}(t)})\right)$$ (7)

calculated using the astrocytic intracellular sodium (Na_ast_), potassium (K_ast_) and hydrogen (H_ast_) concentrations, the extracellular (synaptic) sodium (Na_syn_), potassium (K_syn_) and hydrogen (H_syn_) concentrations, the gas constant, R, and temperature, T. The decision to use a mixture of variable (argument *t*) and constant concentrations arose from considering those ionic concentrations which are low at equilibrium, and thus more highly sensitive to the described fluxes.

The corresponding change intracellular astrocytic glutamate concentration is given by

$$\frac{d}{dt}{Glu}_{ast}(t)=-\frac{{Glu}_{ast}(t)}{\tau_{g}}+\frac{V_{EAAT}(t)}{{Vol}_{ast}}+c$$ (8)

In Eq (9), τ_g_ has been a value to reflect a slow decay rate of glutamate due to enzyme activity and passive diffusion (in relation to rapid synaptic clearance), and a constant c is adjusted to set it to the proposed basal concentration level.

### Astrocytic Na^+^ and synaptic K^+^ dynamics

In addition to Glu^-^, the EAAT cotransports 3Na^+^ and counter-transports 1K^+^, thus we account for changes to astrocytic Na^+^ and extracellular K^+^ by the equations:

$$\frac{d}{dt}Na_{ast}(t)=-\frac{3V_{EAAT}(V_{rev}(t),V_{a})+3V_{ATPase}(t)+V_{NCX}(t)}{{Vol}_{ast}}$$ (9)

$$\frac{d}{dt}K_{syn}(t)=\frac{V_{EAAT}(t)+{2V}_{ATPase}(t)}{Vol_{syn}}+0.5\delta (t-t_{k})$$ (10)

where V_NCX_ denotes the rate of NCX (mM/s) [67] and V_ATPase_ the rate of Na^+^/K^+^ ATPase pump [68], both located on the astrocytic membrane [69]. K^+^_syn_ is also increased in a similar fashion to presynaptic glutamate (5), described by the Dirac delta function. These are described in Eqs (11) and (12) (with parameters detailed in S1 Table)

$$V_{NCX}({Ca}_{ast}(t),{Na}_{ast}(t))=\frac{1}{F}\left[(\bar{I_{NCX}}{\left(\frac{{Na}_{ast}(t)}{{Na}_{syn}}\right)}^{3}\exp\left(\frac{0.5{FV}_{a}}{10^{3}RT}\right)-(\frac{{Ca}_{ast}(t)}{{Ca}_{syn}})\exp\left(\frac{0.5{FV}_{a}}{10^{3}RT}\right)){SA}_{ast}\right]$$ (11)

$$V_{ATPase}(Na_{ast}(t),K_{syn}(t))=P_{ATPAse,max}\left(\frac{{Na}_{ast}{\left(t\right)}^{1.5}}{{Na}_{ast}{\left(t\right)}^{1.5}+K_{Nai}^{1.5}}\right)\;\left(\frac{K_{syn}(t)}{K_{syn}(t)+K_{KE}}\right)\;{SA}_{ast}$$ (12)

As a result of these ionic fluxes, we can approximate the change in astrocytic membrane potential (dV_a_) as

$$C_{a}\frac{dV_{a}(t)}{dt}=F(-V_{ATPase}(t)-V_{EAAT}(t)-V_{NCX}(t))$$ (13)

where C_a_ is astrocytic membrane capacitance (1 Farad).

### Astrocytic IP_3_ and Ca^2+^ activity

We propose two domains of [Ca^2+^] dynamics: one at the soma which is subject to IP3-mediated ER release and SERCA uptake, the other at the perisynaptic process into which ER-originated Ca^2+^ flows and is removed through the membrane by the NCX.

Synaptic glutamate will activate a fraction of G-protein coupled receptors, γ_A_, according to [27]

$$\tau_{A}\frac{d\gamma_{A}(t)}{dt}={-\gamma }_{A}(t)+O_{M}(1-\zeta )Glu_{syn}(t)(1-\gamma_{a}(t))\tau_{A}$$ (14)

using receptor unbinding constant τ_A_ (s), binding rate, O_M_ (mM^−1^ s^−1^), and synaptic transmission efficacy ζ. The activation of these receptors will signal production of IP_3_ via the PLC-β and PLC-δ pathways and degradation of secondary messenger IP_3_ by IP_3_ 3-kinase (IP3K) and inositol polyphosphatase 5-phosphatase (IP5P) as given in [27]:

$$\frac{d}{dt}IP_{3}(t)=J_{\beta }(\gamma_{A}(t))+J_{\delta }({Ca}_{ast}(t),IP_{3}(t))-J_{3K}({Ca}_{ast}(t),IP_{3}(t))-J_{5P}(IP_{3}(t))$$ (15)

where

$$J_{\beta }(\gamma_{A}(t))=O_{\beta }\gamma_{A}(t)$$ (16)

$$J_{\delta }({Ca}_{ast}(t),IP_{3}(t))=O_{\delta }\frac{\kappa_{\delta }}{\kappa_{\delta }+IP_{3}(t)}H({{Ca}_{ast}(t)}^{2},K_{\delta })$$ (17)

$$J_{3K}({Ca}_{ast}(t),IP_{3}(t))=O_{3K}H({{Ca}_{ast}(t)}^{4},K_{D})H(IP_{3}(t),K_{3})$$ (18)

$$J_{5P}(IP_{3}(t))=\Omega_{5P}IP_{3}(t)$$ (19)

In these equations $H(x^{n},K)$ is the Hill function, $\frac{x^{n}}{x^{n}+K^{n}}$, Ca is astrocytic [Ca^2+^] (μM) described below and all other parameters are enumerated in S1 Table.

The concentration of IP_3_ in the astrocytic cytoplasm allows the opening of Ca^2+^ channels located on the ER, which allows an influx of Ca^2+^ into the cytoplasm. Similar to [27], we use Li and Rinzel’s reduced model [70] for soma Ca^2+^ dynamics illustrating the interplay between IP_3_-activated channel flux, J_chan_, leak from the ER, J_leak_, and uptake by the SERCA pumps, J_pump_, given by [27]

$$\frac{d}{dt}{Ca}_{ast,soma}({IP}_{3}(t),{Ca}_{ast,soma}(t),h(t))=J_{chan}(IP_{3}(t),{Ca}_{ast,soma}(t),h(t))+J_{leak}({Ca}_{ast,soma}(t))-J_{SERCA}({Ca}_{ast,soma}(t))$$ (20)

The perisynaptic process compartment Ca^2+^ is given by

$$\frac{d}{dt}{Ca}_{ast,process}({IP}_{3}(t),{Ca}_{ast,soma}(t),h(t),Ca_{ast,process}(t),Na_{ast}(t))=J_{chan}(IP_{3}(t),{Ca}_{ast,soma}(t),h(t))+J_{leak}({Ca}_{ast,process}(t))-\frac{2}{3}V_{NCX}({Ca}_{ast,soma}(t),{Na}_{ast}(t))$$ (21)

where gating variable h is given by,

$$\frac{d}{dt}h(t)=\frac{h_{\infty }({Ca}_{ast}(t),IP_{3}(t))-h(t)}{\tau_{h}({Ca}_{ast}(t),IP_{3}(t))}$$ (22)

where

$$J_{c}({Ca}_{ast}(t),h(t),IP_{3}(t))=\Omega_{C}{m(t)}_{\infty }^{3}{h(t)}^{3}(C_{T}-(1+\rho_{A}){Ca}_{ast}(t))$$ (23)

$$m_{\infty }^{3}({Ca}_{ast}(t),IP_{3}(t))=H(IP_{3}(t),d_{1})H({Ca}_{ast}(t),d_{5})$$ (24)

$$J_{leak}({Ca}_{ast}(t))=\Omega_{L}(C_{T}-(1-\rho_{A}){Ca}_{ast}(t))$$ (25)

$$J_{SERCA}({Ca}_{ast}(t))=O_{p}H({{Ca}_{ast}(t)}^{2},K_{p})$$ (26)

$$h_{\infty }({Ca}_{ast}(t),IP_{3}(t))=\frac{d_{2}(IP3(t)+d_{1})}{d_{2}(IP3(t)+d_{1})+(IP3(t)+d_{3}){Ca}_{ast}(t)}$$ (27)

$$\tau_{h}({Ca}_{ast}(t),IP_{3}(t))=\frac{IP_{3}(t)+d_{3}}{\Omega_{2}(IP_{3}(t)+d_{1})+O_{2}(IP_{3}(t)+d_{3}){Ca}_{ast}(t)}$$ (28)

### Gliotransmission model

The model is designed so that as the astrocytic [Ca^2+^] increases past a threshold C_θ_ concentration (0.5 μM), glutamate is released by the astrocytic into the extra-synaptic compartment. Here we implement a model [27] for describing the fractional increase of readily releasable glutamate vesicles, r_A_, due to each Ca^2+^ transient, which occurs at time t_j_. The fraction of readily releasable vesicles is described by [27]

$$r_{A}(t)=U_{A}x_{A}(t)$$ (29)

where U_A_ is the resting glutamate release probability and x_A_ is the fraction of available vesicles, described according using [27]

$$\tau_{G}\frac{d}{dt}x_{A}(t)=1-x_{A}(t)-r_{A}(t)\delta (t-t_{j})\tau_{G}$$ (30)

where τ_G_ is the glutamate recycling time constant (sec). This will determine the amount of gliotransmitter released, G_rel_ (mM), [27]

$$G_{rel}(t)=\rho_{e}G_{T}r_{A}(t);$$ (31)

where ρ_e_ is the vesicle to extra-synaptic volume fraction and G_T_ is concentration of glutamate in astrocytic vesicles (mM). In our model we vary the concentration of vesicular glutamate according to the following scheme:

$$G_{T}=\rho_{G}G$$ (32)

In this equation, glutamate will increase proportionally with increase of glutamate equilibrium concentration, thus $\rho_{G}=\frac{Glu_{ast,eq}}{Glu_{ast,norm}}$, Glu_ast,norm_ is proposed ‘normal’ astrocytic glutamate concentration (mM), and G is ‘normal’ vesicular glutamate concentration (mM) [27]. Therefore, the change in concentration of glutamate (mM/msec) in the extra-synaptic compartment, Glu_A_, will be given by

$$\tau_{E}\frac{d}{dt}Glu_{A}(t)=-Glu_{A}(t)+G_{rel}(t)\sum_{j}\delta (t-t_{j})\tau_{E}$$ (33)

where τ_E_ is the time constant (msec) for glutamate in this compartment, primarily determined by diffusion.

Glutamate in this extra-synaptic compartment will activate glutamatergic receptors AMPARs and NMDARs according to the following scheme, in which p_ros_AMPA_ and p_ros_NMDA_ indicates the fraction of AMPARs and NMDARs in the ‘open’ state subject to differing binding and unbinding rate constants: μ_1_ and μ_2_ for AMPA, μ_3_ and μ_4_ for NMDA (mM/msec) [71]:

$$\frac{d}{dt}p_{ros,AMPA}(t)=\mu_{1}G_{A}(t)(1-p_{ros,AMPA}(t))-\mu_{2}p_{ros,AMPA}(t)$$ (34)

$$\frac{d}{dt}p_{ros,NMDA}(t)=\mu_{3}G_{A}(t)(1-p_{ros,NMDA}(t))-\mu_{4}p_{ros,NMDA}(t)$$ (35)

The associated NMDA and AMPA current is given by [64]

$$I_{NMDA}(t)=g_{NMDA}Mg(V_{m}(t))p_{ros\_NMDA}(t)(V_{m}(t)-E_{NMDA})$$ (36)

$$I_{AMPA}(t)=g_{AMPA}p_{ros\_AMPA}(t)(V_{m}(t)-E_{AMPA})$$ (37)

where g_NMDA_ and g_AMPA_ are the maximal conductances for NMDA and AMPA currents, respectively, E_NMDA_ and E_AMPA_ are the reversal potentials for NMDA and AMPA currents respectively. The NMDA receptor contains a voltage-dependent magnesium (Mg^2+^) block described by [64]

$$Mg(V_{m}(t))=\frac{1}{1+\frac{\left[Mg\right]}{3.57}exp(-0.06V_{m}(t))}$$ (38)

Thus the resulting astrocyte-induced slow inward current (SIC) can be expressed as [36]

$$I_{sic}(t)=I_{NMDA}(t)+I_{AMPA}(t)$$ (39)

### Postsynaptic neuronal model

The postsynaptic neuronal membrane potential is thus determined as [72]

$$C\frac{dV_{m}(t)}{dt}=-I_{Na}(t)-I_{K}(t)-I_{sic}(t)-I_{leak}(t)-I_{syn}(t)$$ (40)

where capacitance, C, is 1μF/cm^2^, V_m_ is membrane potential (mV), I_sic_ is the slow-inward current (μA /cm^2^) described by Eq (39) and I_syn_ denotes the synaptic currents (μA/cm^2^) (Eqs (36 and 37) in response to Glu_syn_). I_Na_, I_K_, I_leak_ describe the Na^+^, K^+^ and leak currents (μA/cm^2^), respectively, and are described by [73]

$$I_{Na}\;(t)=g_{Na}{m(t)}_{\infty }^{3}b(t)(V_{m}(t)-E_{Na});$$ (41)

$$I_{K}(t)=g_{k}{n(t)}^{4}(V_{m}(t)-E_{K});$$ (42)

$$I_{leak}(t)=g_{leak}(V_{m}(t)-E_{leak})$$ (43)

where g_Na_, g_K_ and g_leak_ denote conductances of sodium, potassium channels, and leak (mS/cm^2^), and E_Na_, E_K_ and E_leak_ are the reversal potentials (mV) for sodium, potassium and leak respectively. The following equations describe the kinetics of the neuronal Na^+^ channel [73]:

$${m(t)}_{\infty }=\frac{1}{1+exp(-\frac{V_{m}(t)+30}{9.5})};$$ (44)

$$\tau_{b}(t)=0.1+\frac{0.75}{1+exp(-\frac{V_{m}(t)+40.5}{-6})};$$ (45)

$$\frac{db(t)}{dt}=\frac{{b(t)}_{\infty }-b(t)}{\tau_{b}(t)}$$ (46)

$$b_{\infty }=\frac{1}{1+exp(\frac{V_{m}(t)+45}{7})};$$ (47)

The following equations describe the dynamics of the neuronal K^+^ channel [73]:

$$\tau_{n}(t)=0.1+\frac{0.5}{1+exp(\frac{V_{m}(t)+27}{15})}$$ (48)

$$\frac{dn(t)}{dt}=\frac{{n(t)}_{\infty }-n(t)}{\tau_{n}(t)}$$ (49)

$${n(t)}_{\infty }=\frac{1}{1+exp(-\frac{V_{m}(t)+35}{10})}$$ (50)

### Simulations

The above model was constructed and a 10 Hz presynaptic regular spike train was used to represent the firing activity of the presynaptic neuron. To ascertain the significance of astrocytic glutamate concentration, three basal intracellular glutamate concentrations were compared: 1.5mM, 5mM and 10mM. The first and second of these values are within the normal physiological range for astrocytic glutamate of 0.5–5mM [57] and the third value was chosen as a prediction for pathological conditions. Due to multiple time scales in the system, we considered two simulations: the first considers the neuron-to-astrocyte communication, the second considers the astrocyte-to-neuron interaction. The neuron-to-astrocyte simulation uses the forward Euler numerical integration scheme with 1 ms time step, where a 10 Hz neuronal spike train results in a slower astrocytic response. This response is then interpolated in order to increase the time step to 0.01 ms to simulate the faster neuronal response. Both time scales are numerically integrated using the forward Euler method with MATLAB R2013. We considered each step of the model in three different cases where only the equilibrium astrocytic glutamate concentration differs.

## Supporting information

S1 Text Fitting of voltage-dependent GLT-1 (EAAT2) transporter current to experimental data. Description of the process for manipulating EAAT current experimental data to be used for EAAT uptake rate.
(DOCX)

S1 Fig Ionic stability diagram. Stability diagram of astrocytic Na^+^ and synaptic K^+^ and Glu^-^ activity on frequency of periodic presynaptic firing activity under different baseline astrocytic level [Glu]_ast,eq_.
(TIF)

S1 Table Table of parameters. List of values for the parameters used in the model simulations.
(DOCX)
