# Supplementary material for: A computational study of astrocytic glutamate influence on post-synaptic neuronal excitability
Source: PLoS Comput Biol. 2018 Apr 16;14(4):e1006040. doi: 10.1371/journal.pcbi.1006040 (PMC5919689; doi:10.1371/journal.pcbi.1006040)
Supplement: S1 Text — Description of the process for manipulating EAAT current experimental data to be used for EAAT uptake rate. (DOCX) [file pcbi.1006040.s001.docx]

**S1 Text. Fitting of voltage-dependent GLT-1 (EAAT2) transporter current to experimental data.**


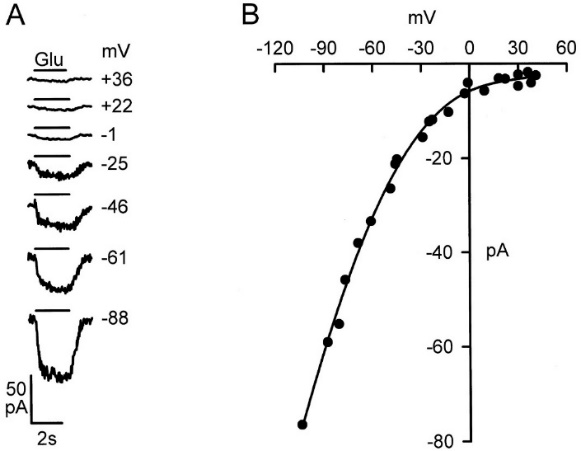


Fig 1. Original Data: Caption taken from [1] “Voltage dependence of glutamate-evoked currents in CHO cells expressing GLT-1. A, Currents evoked by 100 μm glutamate (black bars) in a cell clamped to the potentials shown by each trace. B, Peak glutamate-evoked current as a function of voltage for the cell in A; similar results were obtained from seven cells” Levy et al. 1998

This data was chosen as it demonstrated the voltage-dependence of the GLT-1 (EAAT2) transporter, isolated from synaptic interference.

1. ***Extending the curve***

We started by manipulating the data, instead of the x axis displaying membrane potential ‘V’, instead we shift the curve so that I is now given as a function of ($V-V_{rev}$), and assuming that the point of zero flux is given by

$$I\left( V-V_{rev} \right)=0$$

The value of $V_{rev}$ was chosen using the solution concentrations (Table 1) of the original experiment.

Solutions. *For measuring forward uptake currents, the cells were clamped with electrodes filled with solution containing (in mm) 140 KCl, 0.5 CaCl_2_, 5 Na_2_-EGTA, 10 HEPES, 2 MgCl_2_, and 1 Na_2_ATP, pH 7.0, in external solution containing (in mm) 140 NaCl, 2.5 KCl, 10 HEPES, 2 MgCl_2_, 2.5 CaCl_2_, 1 Na_2_HPO_4_, and 10 glucose, pH 7.4. [Levy et al. 1998]*

$$V_{\mathrm{rev}}=\frac{\mathrm{RT}}{2F}\ln\left( \left( \frac{{{[Na}^{+}]}_{\mathrm{ex}}}{{{[Na}^{+}]}_{\mathrm{in}}} \right)^{3}\frac{{{[H}^{+}]}_{\mathrm{ex}}}{{{[H}^{+}]}_{\mathrm{in}}}\frac{{{[Glu}^{-}]}_{\mathrm{ex}}}{{{[Glu}^{-}]}_{\mathrm{in}}}\frac{{{[K}^{+}]}_{\mathrm{in}}}{{{[K}^{+}]}_{\mathrm{ex}}} \right) \approx131.2 mV$$

This adds the point$\left( I,V \right) =(0,131.2)$to the original data (Fig 2).

**Table 1: Ionic Concentrations (mM) used in original experiment [1]**

| Ion | Intracellular | | Extracellular | |
| --- | --- | --- | --- | --- |
|  | **Ionic Compound(s)** | **Concentration** | **Ionic Compound(s)** | **Concentration** |
| Na+ | 5 Na_2_-EGTA  1 Na_2_ATP | 12 (=5x2 + 1x2) | 140 NaCl  1 Na_2_ HPO_4_ | 142 (=140 + 1x2) |
| K+ | 140 KCl | 140 | 2.5 KCl | 2.5 |
| H+ | pH 7.0 | 10^-7^ | pH 7.4 | 10^-7.4^ |
| Glu- |  | 0.2 |  | 0.1 |

1. ***Scaling the current***

The data recorded [1] illustrates the relationship between membrane potential and current from the EAAT transporters under saturating concentrations of glutamate. We wish to propose an adaptable model to illustrate the maximal current of the EAAT2 where the density of transporters is especially high, at the glutamatergic synapses [2], but the surface area is much smaller than the cell surface area recorded [1]. We therefore require the current in their experimental data to be converted to current density, which can therefore be adapted for our use. Although not explicitly stated, from a previous work by the team [3] they have demonstrated that where an identical cell type is clamped at 60mV for the current of ~-30pA to be evoked [1], the cell is likely to have a radius of ~21μm. We use this information to deduce that the surface area of the cell investigated is ~1764π x 10^-12^ m^2^ and thus the current measured can be scaled using these values.

Therefore for our purposes:

$$I\to\frac{I}{A\times D} ={\frac{I}{1764\pi\times{10}^{-12}}\mathrm{pA}}/{m^{2}}$$

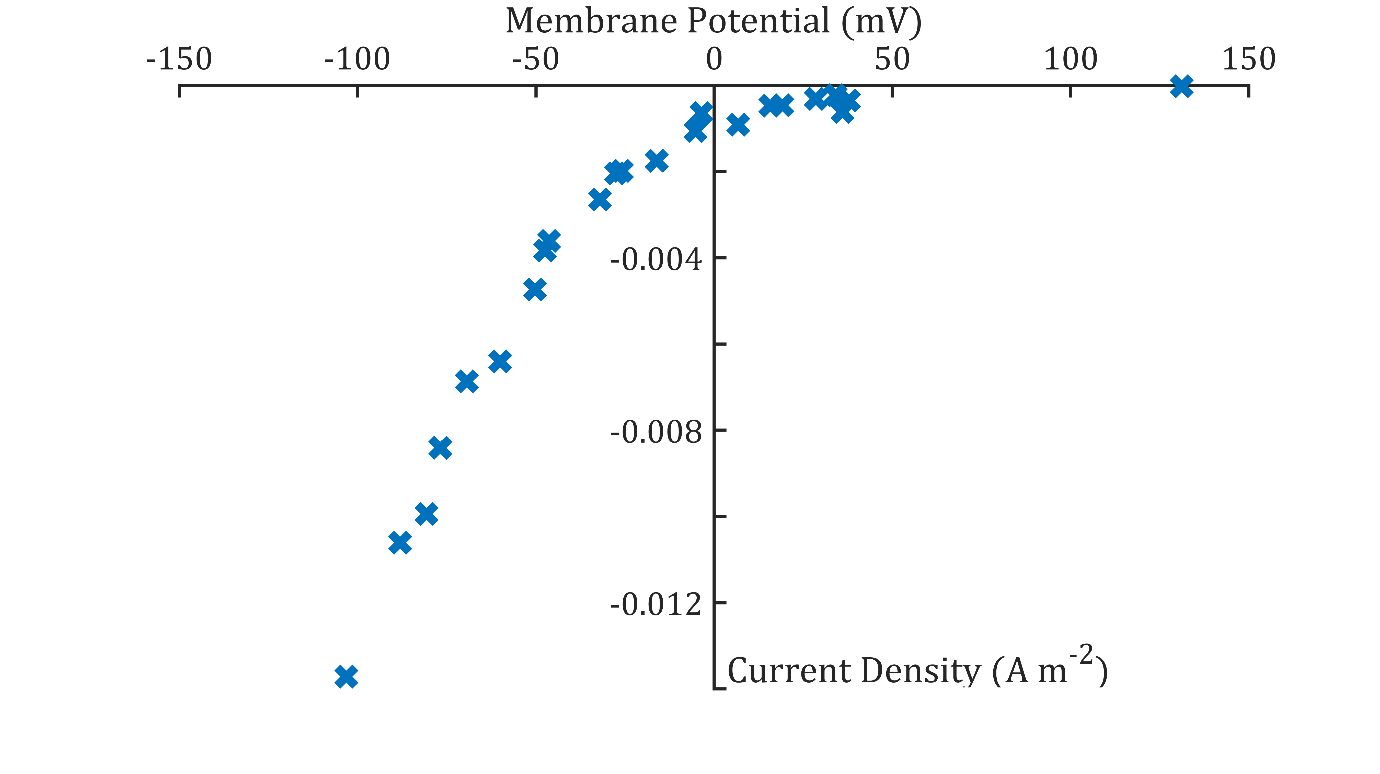


Fig 2. Original membrane potential-dependent data including point of zero flux and current converted to current density

We require a description of I as a function of (V-V_rev_) so that I may alter according to a variable driving force, therefore Fig 2 is translated in the x-direction so that the point of zero flux is given at ((V-V_rev_), I)=(0,0), as illustrated in Fig 3.


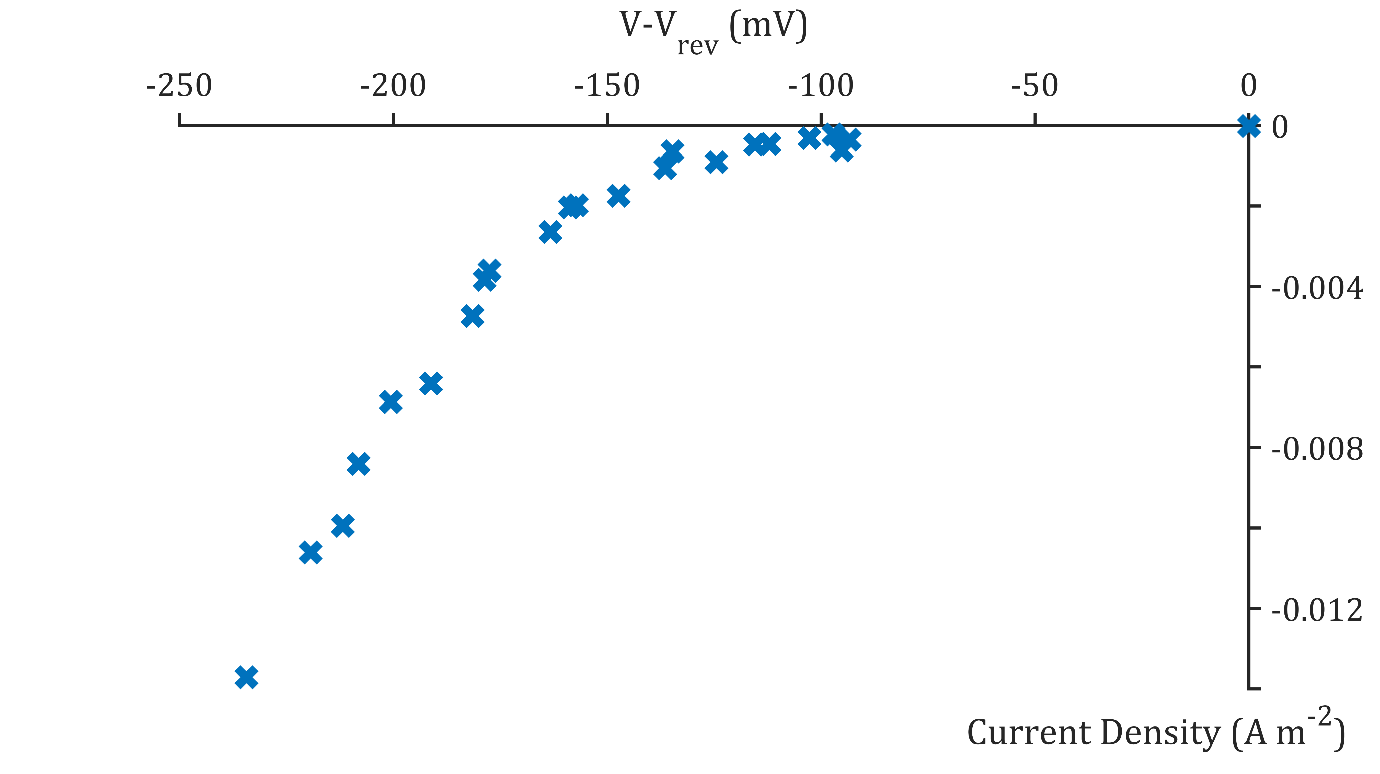


Fig 3. New data illustrating the relationship between driving force (V-V_rev_) and current density (I)

1. ***Curve Fitting***

Recognising the shape of the curve excludes a linear or quadratic relationship, we firstly fitted the data points of the graph to a cubic polynomial however this resulted in a high $\sum error^{2}$and this made assumptions that the curve was symmetric about the point of zero flux, which is unlikely. We next consider fitting the curve to an exponential function.

If we write the above function as

$$I=\alpha e^{\beta\left( V-V_{rev} \right)}$$

Applying logs:

$$\ln(I)=\ln\alpha+\ln e^{\beta\left( V-V_{\mathrm{rev}} \right)}=\ln\alpha+\beta\left( V-V_{\mathrm{rev}} \right)$$

Using our manipulated data points from experimental observations [1] allows us to determine$\alpha$and$\beta$. We plot -ln(-I) against (V-V_rev_) (Fig 4) and our parameters $\alpha$and$\beta$ can be given as the exponent of the y intercept and the gradient of the fitted line, respectively.


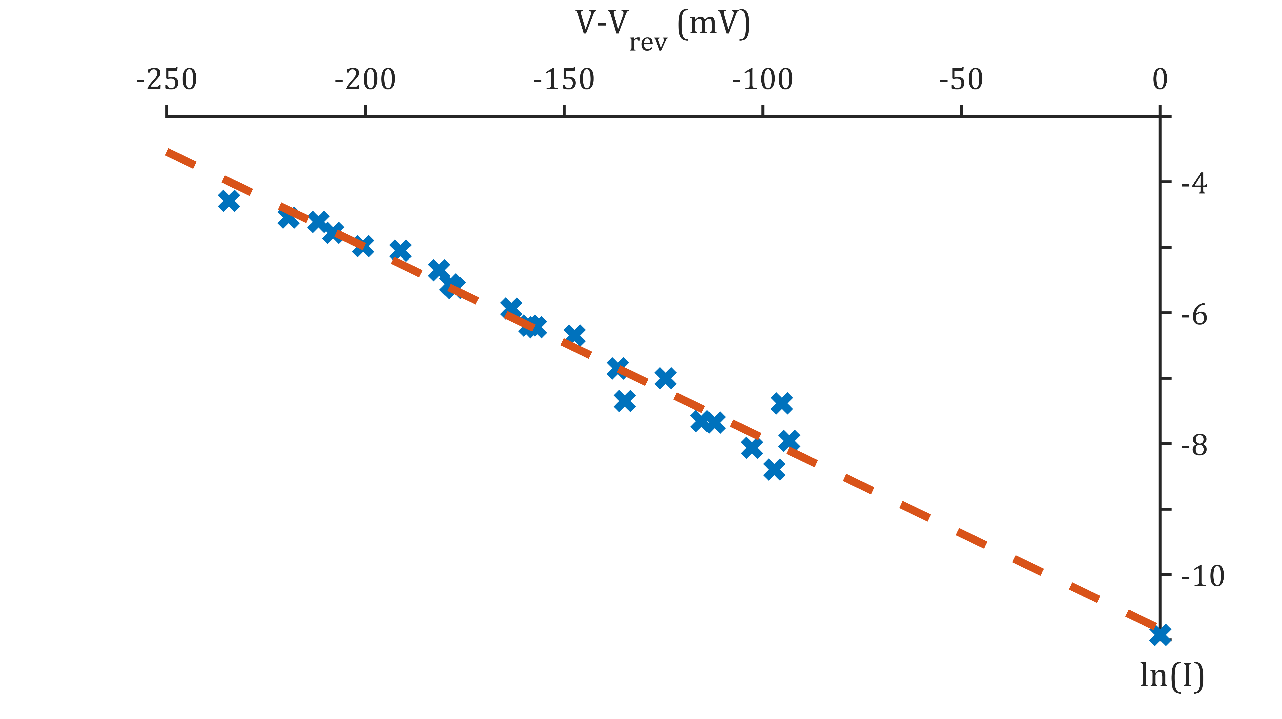


Fig 4. Log-linear plot of the data from Fig 1**.** This is used to find the $\alpha$and$\beta$ parameters from our model equation. (Red- fitted line: $\alpha=1.9767\times{10}^{-5}A m^{-2} , \beta=0.0292 \mathrm{mV}^{-1}$)

The extension of the curve past the determined data points allows us to define the required relationship as

$$J=-\alpha\left( e^{-\beta\left( V-V_{\mathrm{rev}} \right)} \right)$$

where$\alpha=1.9767\times{10}^{-5}A m^{-2} , \beta=0.0292 \mathrm{mV}^{-1}.$This fitted curve is plotted in Fig 5.


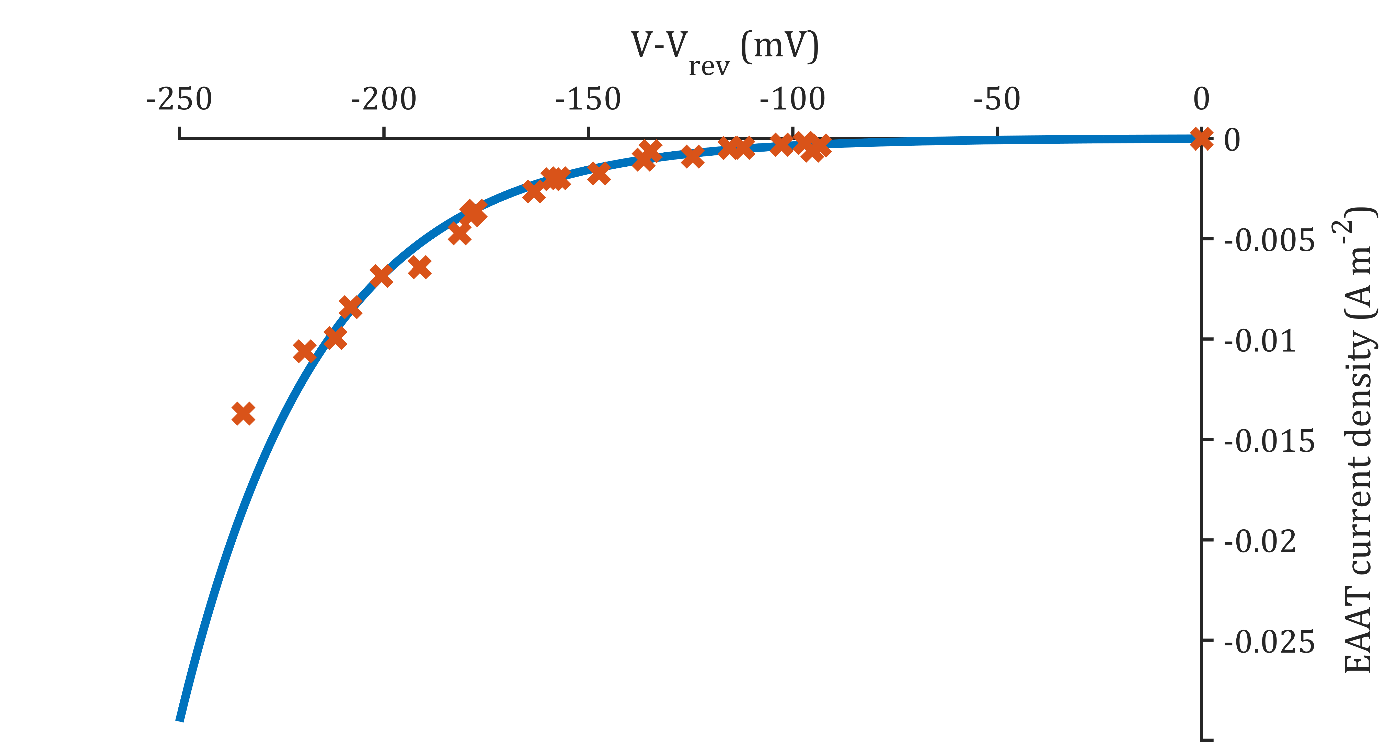


Fig 5. Final EAAT model function: where driving force is plotted against current density

1. ***Comparison with astrocyte-based synaptic transporter currents (STCs)***

We applied our model to the experimental procedure solutions of Bergles and Jahr (1997) [4], where the astrocytic transporter currents were measured at four discrete points: in response to glutamate applications of 10μM, 100μM, 1mM and 10mM. We used their ionic concentrations of intracellular K^+^ and H^+^ of 130mM and 10^-7.2^mM, respectively, and extracellular Na^+^, K^+^ and H^+^ of 135mM, 4.5mM and 10^-7.2^ mM, respectively. We assumed the intracellular Na+ and Glu- to be 0.01mM and 1mM, respectively, in order for our model to operate and compared the magnitude of current response to the experimental data (Fig 6). We find a close resemblance to the experimental-evoked STCs using our model at physiological concentrations (0.01-1mM) which is over the range of interest (Fig 6 inset).


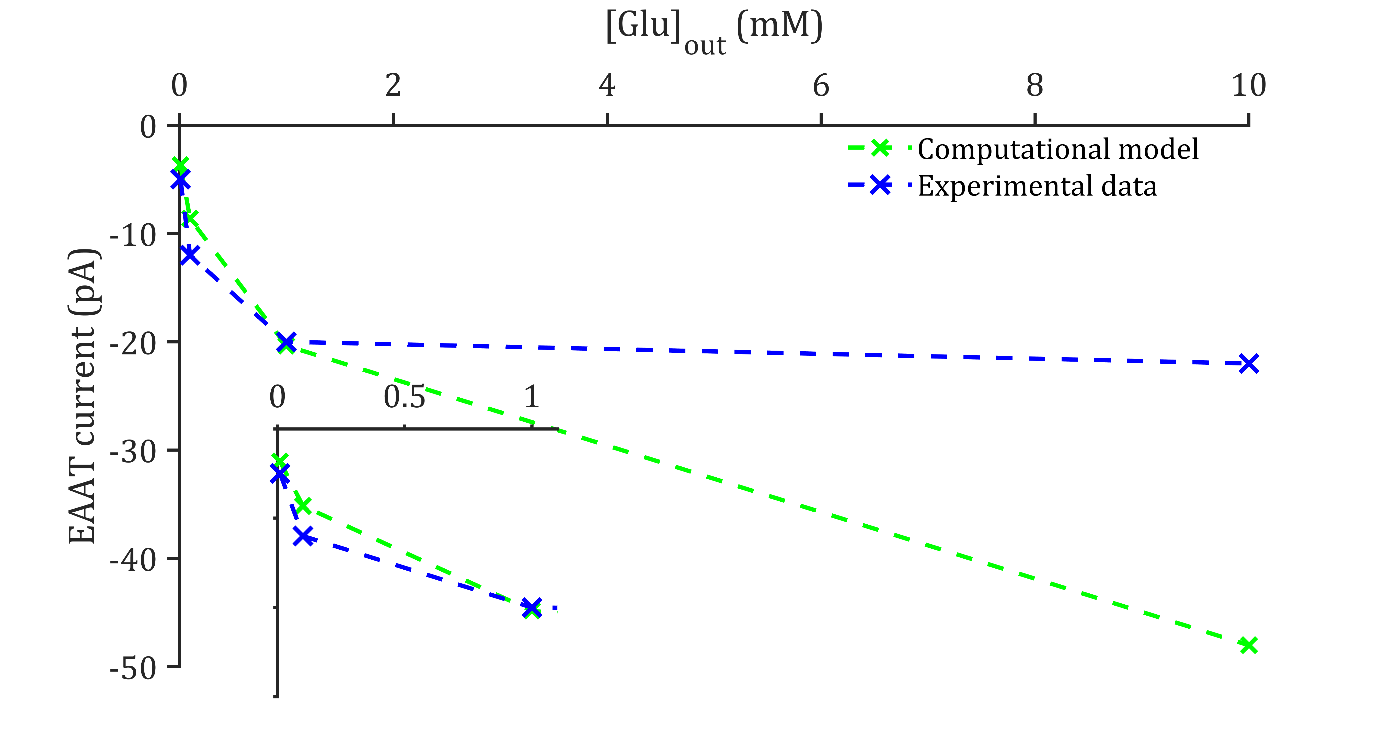


Fig 6. Validation of model with experimental synaptic currents [4] (Inset: Range of interest)

References

1. Levy L, Warr O, Attwell D. Stoichiometry of the glial glutamate transporter GLT-1 expressed inducibly in a Chinese hamster ovary cell line selected for low endogenous Na^+^-dependent glutamate uptake. J Neurosci 1998 Dec 1; 18(23):9620-9628.
2. Danbolt N. Glutamate uptake. Prog Neurobiol 2001; 65(1):1-105.
3. Levy LM, Attwell D, Hoover F, Ash JF, Bjørås M, Danbolt NC. Inducible expression of the GLT-1 glutamate transporter in a CHO cell line selected for low endogenous glutamate uptake. FEBS Lett 1998; 422(3):339-342.
4. Bergles, DE. & Jahr, CE. Synaptic activation of glutamate transporters in hippocampal astrocytes. Neuron 1997, 19(6), 1297-1308.
