# Supplementary figures and images for: A computational study of astrocytic glutamate influence on post-synaptic neuronal excitability
Source: PLoS Comput Biol. 2018 Apr 16;14(4):e1006040. doi: 10.1371/journal.pcbi.1006040 (PMC5919689; doi:10.1371/journal.pcbi.1006040)

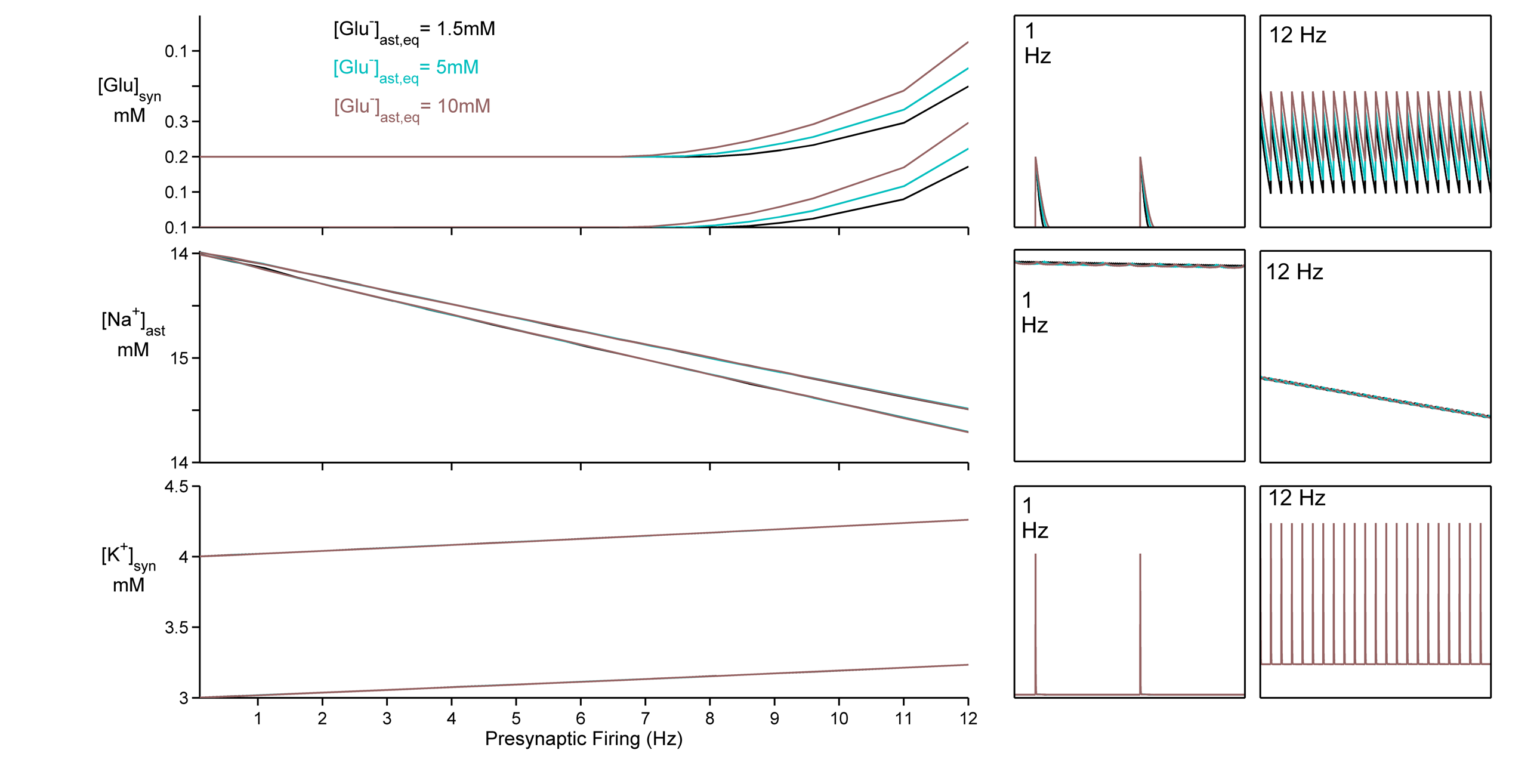

Supplement: S1 Fig — Stability diagram of astrocytic Na+ and synaptic K+ and Glu- activity on frequency of periodic presynaptic firing activity under different baseline astrocytic level [Glu]ast,eq. (TIF) [file pcbi.1006040.s002.tif]
