# Supplementary material for: A computational study of astrocytic glutamate influence on post-synaptic neuronal excitability
Source: PLoS Comput Biol. 2018 Apr 16;14(4):e1006040. doi: 10.1371/journal.pcbi.1006040 (PMC5919689; doi:10.1371/journal.pcbi.1006040)
Supplement: S1 Table — List of values for the parameters used in the model simulations. (DOCX) [file pcbi.1006040.s003.docx]

S1 Table: Table of parameter values

| Parameter | Description | Source | Value | Unit |
| --- | --- | --- | --- | --- |
| $\boldsymbol{Y}_{\boldsymbol{rel}}$ | Synaptic [Glu] released | $\boldsymbol{[27]}$ | $\mathbf{0.1}$ | $\mathbf{mM}$ |
| $\mathbf{F}$ | Faraday’s constant | $\boldsymbol{\sim}$ | $\mathbf{96480}$ | $\mathbf{C mo}\mathbf{l}^{\mathbf{-1}}$ |
| $\mathbf{Vo}\mathbf{l}_{\mathbf{syn}}$ | Volume of the synaptic compartment | * | $\boldsymbol{1}\boldsymbol{0}^{\boldsymbol{-18}}$ | $\mathbf{L}$ |
| $\mathbf{Vo}\mathbf{l}_{\mathbf{ast}}$ | Volume of the perisynaptic process compartment | ** | $\boldsymbol{3.76\times1}\boldsymbol{0}^{\boldsymbol{-17}}$ | $\mathbf{L}$ |
| $\boldsymbol{\alpha}$ | EAAT current fitting parameter | S1 | $\boldsymbol{0.0002}$ | $\mathbf{A}\mathbf{m}^{\mathbf{-2}}$ |
| $\boldsymbol{\beta}$ | EAAT current fitting parameter | S1 | $\boldsymbol{0.0288}$ | $\mathbf{m}\mathbf{V}^{\mathbf{-1}}$ |
| $\left[ \mathbf{N}\mathbf{a}^{\mathbf{+}} \right]_{\mathbf{syn}}$ | Extracellular/synaptic [Na^+^] | $\boldsymbol{[69]}$ | $\mathbf{140}$ | $\mathbf{mM}$ |
| $\left[ \mathbf{K}^{\mathbf{+}} \right]_{\mathbf{ast}}$ | Astrocytic intracellular [K^+^] | $\boldsymbol{[69]}$ | $\mathbf{100}$ | $\mathbf{mM}$ |
| $\left[ \mathbf{H}^{\mathbf{+}} \right]_{\mathbf{ast}}$ | Astrocytic intracellular[H^+^] | $\left[ \boldsymbol{38} \right]$ | $\boldsymbol{60}$ | $\mathbf{nM}$ |
| $\left[ \mathbf{H}^{\mathbf{+}} \right]_{\mathbf{syn}}$ | Extracellular/synaptic [H^+^] | $\left[ \boldsymbol{38} \right]$ | $\mathbf{40}$ | $\mathbf{nM}$ |
| $\mathbf{R}$ | Gas constant | $\boldsymbol{[69]}$ | $\mathbf{8.3145}$ | $\mathbf{J}\mathbf{K}^{\mathbf{-1}}\mathbf{mo}\mathbf{l}^{\mathbf{-1}}$ |
| $\mathbf{T}$ | Temperature | $\boldsymbol{[69]}$ | $\mathbf{310}$ | $\mathbf{K}$ |
| $\boldsymbol{\tau}_{\mathbf{g}}$ | Astrocytic intracellular glutamate clearance | $\boldsymbol{*}$ | $\mathbf{200}$ | $\mathbf{ms}$ |
| $\mathbf{I}_{\mathbf{NCX}}$ | Maximum NCX current | [67] | $\boldsymbol{1}$ | $\mathbf{A}\mathbf{m}^{\mathbf{-2}}$ |
| $\mathbf{S}\mathbf{A}_{\mathbf{ast}}$ | Perisynaptic process surface area | *** | $\boldsymbol{2.8274\times1}\boldsymbol{0}^{\boldsymbol{-13}}$ | $\boldsymbol{m}^{\boldsymbol{2}}$ |
| $\mathbf{P}_{\mathbf{ATPase,max}}$ | Maximum NaK-ATPase pump rate | [68] | $\mathbf{1}\mathbf{0}^{\mathbf{-6}}$ | $\mathbf{mol}\mathbf{m}^{\mathbf{-2}}$ |
| $\mathbf{K}_{\mathbf{Nai}}$ | $\mathbf{NaK-ATPase}$ affinity for$\mathbf{N}\mathbf{a}^{\mathbf{+}}$ | [68] | $\mathbf{10}$ | $\mathbf{mM}$ |
| $\mathbf{K}_{\mathbf{KE}}$ | NaK-ATPase affinity for $\mathbf{K}^{\mathbf{+}}$ | [68] | $\mathbf{1.5}$ | $\mathbf{mM}$ |
| $\boldsymbol{\tau}_{\mathbf{A}}$ | GPCR agonist unbinding rate | $\left[ \boldsymbol{27} \right]$ | $\mathbf{0.55}$ | $\mathbf{s}$ |
| $\mathbf{O}_{\mathbf{M}}$ | GPCR agonist binding rate | $\left[ \boldsymbol{27} \right]$ | $\boldsymbol{0.3\times}\mathbf{10}^{\mathbf{3}}$ | $\boldsymbol{\mu}\mathbf{M}^{\mathbf{-1}} \mathbf{s}^{\mathbf{-1}}$ |
| $\boldsymbol{\zeta}$ | Efficacy of synaptic transmission | $\left[ \boldsymbol{27} \right]$ | $\mathbf{0.75}$ | $\boldsymbol{\sim}$ |
| $\mathbf{O}_{\boldsymbol{\beta}}$ | Max. rate of IP_3_ production by PLCβ | $\left[ \boldsymbol{27} \right]$ | $\mathbf{1}$ | $\boldsymbol{\mu M}\mathbf{s}^{\mathbf{-1}}$ |
| $\mathbf{O}_{\boldsymbol{\delta}}$ | Max. rate of IP_3_ production by PLCδ | $\left[ \boldsymbol{27} \right]$ | $\mathbf{0.05}$ | $\boldsymbol{\mu M}\mathbf{s}^{\mathbf{-1}}$ |
| $\boldsymbol{\kappa}_{\boldsymbol{\delta}}$ | Inhibiting IP_3_ affinity of PLCδ | $\left[ \boldsymbol{27} \right]$ | $\mathbf{1}$ | $\boldsymbol{\mu M}$ |
| $\mathbf{K}_{\boldsymbol{\delta}}$ | Ca^2+^ affinity of PLCδ | $\left[ \boldsymbol{27} \right]$ | $\mathbf{0.5}$ | $\boldsymbol{\mu M}$ |
| $\mathbf{O}_{\mathbf{3K}}$ | Max. rate of IP_3_ degradation by IP_3-3K_ | $\left[ \boldsymbol{27} \right]$ | $\mathbf{4.5}$ | $\boldsymbol{\mu M}\mathbf{s}^{\mathbf{-1}}$ |
| $\mathbf{K}_{\mathbf{D}}$ | Ca^2+^ affinity of IP_3-3K_ | $\left[ \boldsymbol{27} \right]$ | $\mathbf{0.5}$ | $\boldsymbol{\mu M}$ |
| $\mathbf{K}_{\mathbf{3}}$ | IP_3_ affinity of IP_3-3K_ | $\left[ \boldsymbol{27} \right]$ | $\mathbf{1}$ | $\boldsymbol{\mu M}$ |
| $\boldsymbol{\Omega}_{\mathbf{5P}}$ | Max. rate of degradation of IP_3_ by IP-5P | $\left[ \boldsymbol{27} \right]$ | $\mathbf{0.1}$ | $\mathbf{s}^{\mathbf{-1}}$ |
| $\boldsymbol{\Omega}_{\mathbf{C}}$ | Max rate of Ca^2+^ release from IP_3_Rs | $\left[ \boldsymbol{27} \right]$ | $\mathbf{6}$ | $\mathbf{s}^{\mathbf{-1}}$ |
| $\mathbf{C}_{\mathbf{T}}$ | Total ER Ca^2+^ content | $\left[ \boldsymbol{27} \right]$ | $\mathbf{2}$ | $\boldsymbol{\mu M}$ |
| $\boldsymbol{\rho}_{\mathbf{A}}$ | ER:cytoplasm volume ratio | $\left[ \boldsymbol{27} \right]$ | $\mathbf{0.18}$ | $\boldsymbol{\sim}$ |
| $\mathbf{d}_{\mathbf{1}}$ | IP_3_ binding affinity to IP_3_Rs | $\left[ \boldsymbol{27} \right]$ | $\mathbf{0.13}$ | $\boldsymbol{\mu M}$ |
| $\mathbf{d}_{\mathbf{2}}$ | Inact. Ca^2+^ binding affinity to IP_3_Rs | $\left[ \boldsymbol{27} \right]$ | $\mathbf{1.05}$ | $\boldsymbol{\mu M}$ |
| $\mathbf{d}_{\mathbf{3}}$ | IP_3_ binding affinity to IP_3_Rs | $\left[ \boldsymbol{27} \right]$ | $\mathbf{0.9434}$ | $\boldsymbol{\mu M}$ |
| $\mathbf{d}_{\mathbf{5}}$ | Act. Ca^2+^ binding affinity to IP_3_Rs | $\left[ \boldsymbol{27} \right]$ | $\mathbf{0.08}$ | $\boldsymbol{\mu M}$ |
| $\boldsymbol{\Omega}_{\mathbf{L}}$ | Max. Ca^2+^ leak rate | $\left[ \boldsymbol{27} \right]$ | $\mathbf{0.1}$ | $\mathbf{s}^{\mathbf{-1}}$ |
| $\mathbf{O}_{\mathbf{P}}$ | Max Ca^2+^ uptake rate SERCA | $\left[ \boldsymbol{27} \right]$ | $\mathbf{0.9}$ | $\boldsymbol{\mu M}\mathbf{s}^{\mathbf{-1}}$ |
| $\mathbf{K}_{\mathbf{P}}$ | Ca^2+^ affinity of SERCA pumps | $\left[ \boldsymbol{27} \right]$ | $\mathbf{0.05}$ | $\boldsymbol{\mu M}$ |
| $\mathbf{O}_{\mathbf{2}}$ | Inact. Ca^2+^ binding rate | $\left[ \boldsymbol{27} \right]$ | $\mathbf{0.2}$ | $\boldsymbol{\mu M}\mathbf{s}^{\mathbf{-1}}$ |
| $\boldsymbol{\Omega}_{\mathbf{2}}$ | Product of inact. Ca^2+^ affinity and binding rates | $\left[ \boldsymbol{27} \right]$ | $\mathbf{0.21}$ | $\boldsymbol{\mu}\mathbf{M}^{\mathbf{2}}\mathbf{s}^{\mathbf{-1}}$ |
| $\mathbf{C}_{\boldsymbol{\theta}}$ | Ca^2+^ threshold for gliotransmission | $\left[ \boldsymbol{27} \right]$ | $\mathbf{0.5}$ | $\boldsymbol{\mu M}$ |
| $\mathbf{U}_{\mathbf{A}}$ | Resting glutamate release probability | $\left[ \boldsymbol{27} \right]$ | $\mathbf{0.6}$ | $\boldsymbol{\sim}$ |
| $\boldsymbol{\tau}_{\mathbf{G}}$ | Glutamate recycling time constant | $\left[ \boldsymbol{27} \right]$ | $\mathbf{1.66}$ | $\mathbf{s}$ |
| $\boldsymbol{\rho}_{\mathbf{e}}$ | Vesicular versus mixing volume ratio | $\left[ \boldsymbol{27} \right]$ | $\boldsymbol{6.5\times}\mathbf{10}^{\mathbf{-4}}$ | $\boldsymbol{\sim}$ |
| $\mathbf{G}_{\mathbf{T}}$ | Total vesicular (astrocyte) glutamate  concentration | $\left[ \boldsymbol{27} \right]$ | $\mathbf{250}$ | $\mathbf{mM}$ |
| $\boldsymbol{\tau}_{\mathbf{e}}$ | Glutamate clearance time constant | $\left[ \boldsymbol{27} \right]$ | $\mathbf{200e-3}$ | $\mathbf{s}$ |
| $\mathbf{u}_{\mathbf{1}}$ | AMPA forward (binding) rate constant | $\left[ \boldsymbol{71} \right]$ | $\mathbf{1.1}$ | $\mathbf{m}\mathbf{M}^{\mathbf{-1}}\mathbf{m}\mathbf{s}^{\mathbf{-1}}$ |
| $\mathbf{u}_{\mathbf{2}}$ | AMPA backward (unbinding) rate constant | $\left[ \boldsymbol{71} \right]$ | $\mathbf{190}$ | $\mathbf{m}\mathbf{M}^{\mathbf{-1}}\mathbf{m}\mathbf{s}^{\mathbf{-1}}$ |
| $\mathbf{u}_{\mathbf{3}}$ | NMDA forward (binding) rate constant | $\left[ \boldsymbol{71} \right]$ | $\mathbf{7.2e-2}$ | $\mathbf{m}\mathbf{M}^{\mathbf{-1}}\mathbf{m}\mathbf{s}^{\mathbf{-1}}$ |
| $\mathbf{u}_{\mathbf{4}}$ | NMDA backward (unbinding) rate constant | $\left[ \boldsymbol{71} \right]$ | $\mathbf{6.6}$ | $\mathbf{m}\mathbf{M}^{\mathbf{-1}}\mathbf{m}\mathbf{s}^{\mathbf{-1}}$ |
| $\mathbf{g}_{\mathbf{AMPA}}$ | Max. AMPA conductance | $\left[ \boldsymbol{71} \right]$ | $\mathbf{0.0145}$ | $\mathbf{mS c}\mathbf{m}^{\mathbf{-2}}$ |
| $\mathbf{g}_{\mathbf{NMDA}}$ | Max. NMDA conductance | $\left[ \boldsymbol{71} \right]$ | $\mathbf{0.026}$ | $\mathbf{mS c}\mathbf{m}^{\mathbf{-2}}$ |
| $\left[ \mathbf{Mg} \right]$ | Extracellular [Mg^2+^] | $\left[ \boldsymbol{71} \right]$ | $\mathbf{1}$ | $\mathbf{mM}$ |
| $\mathbf{E}_{\mathbf{NMDA}}$ | Reversal potentials for NMDA | $\left[ \boldsymbol{71} \right]$ | $\mathbf{0}$ | $\mathbf{mV}$ |
| $\mathbf{E}_{\mathbf{AMPA}}$ | Reversal potentials for AMPA | $\left[ \boldsymbol{71} \right]$ | $\mathbf{0}$ | $\mathbf{mV}$ |
| $\mathbf{g}_{\mathbf{Na}}$ | Max. conductance Na^+^ Voltage-Gated Channels | $\left[ \boldsymbol{73} \right]$ | $\mathbf{45}$ | $\mathbf{mS c}\mathbf{m}^{\mathbf{-2}}$ |
| $\mathbf{g}_{\mathbf{K}}$ | Max. conductance K^+^ Voltage-Gated Channel | $\left[ \boldsymbol{73} \right]$ | $\mathbf{18}$ | $\mathbf{mS c}\mathbf{m}^{\mathbf{-2}}$ |
| $\mathbf{g}_{\mathbf{L}}$ | Max. conductance of leak channels | $\left[ \boldsymbol{73} \right]$ | $\mathbf{0.05}$ | $\mathbf{mS c}\mathbf{m}^{\mathbf{-2}}$ |
| $\mathbf{E}_{\mathbf{leak}}$ | Reversal Potential of leak | $\left[ \boldsymbol{73} \right]$ | $\mathbf{-70}$ | $\mathbf{mV}$ |
| $\mathbf{E}_{\mathbf{K}}$ | Reversal Potential K^+^ | $\left[ \boldsymbol{73} \right]$ | $\mathbf{-90}$ | $\mathbf{mV}$ |
| $\mathbf{E}_{\mathbf{Na}}$ | Reversal Potential Na^+^ | $\left[ \boldsymbol{73} \right]$ | $\mathbf{55}$ | $\mathbf{mV}$ |
| $\mathbf{C}$ | Postsynaptic capacitance | $\left[ \boldsymbol{73} \right]$ | $\mathbf{1}$ | 1$\boldsymbol{\mu F c}\mathbf{m}^{\mathbf{-2}}$ |

*Volume of synapse taken as approx. 1/10 volume of perisynaptic process

**Volume of perisynaptic process given as difference between two cylinders, radiuses 500x10^-9^ m and 300x10^-9^ m, both length 300x10^-9^ m

*** Surface area of perisynaptic process taken as cylinder of radius 50x10^-9^m and length 300x10^-9^ m
